# Security Audit of IoT Device Networks: A Reproducible Machine Learning Framework for Threat Detection and Performance Benchmarking

**DOI:** 10.3390/s25247519

**Published:** 2025-12-11

**Authors:** Aigul Shaikhanova, Oleksandr Kuznetsov, Aizhan Tokkuliyeva, Kamil Ayapbergenov, Satiev Olzhas, Tlepov Danir

**Affiliations:** 1 Department of Information Security, L.N. Gumilyov Eurasian National University, 2, Satpayeva St., Astana 010000, Kazakhstan; shaikhanova_ak@enu.kz; 2 Department of Theoretical and Applied Sciences, eCampus University, Via Isimbardi 10, 22060 Novedrate, Italy; 3 Department of Intelligent Software Systems and Technologies, School of Computer Science and Artificial Intelligence, V.N. Karazin Kharkiv National University, 4 Svobody Sq., 61022 Kharkiv, Ukraine; 4 Limited Liability Company “TSARKA R&D”, 51/1 Kabanbai Batyr St., Astana 010000, Kazakhstan; akamil@cert.kz (K.A.); os@cert.kz (S.O.); tdanir@cert.kz (T.D.)

**Keywords:** Internet of Things security, intrusion detection systems, network traffic analysis, ensemble learning, security audit, cybersecurity

## Abstract

**Highlights:**

**What are the main findings?**

- Ensemble learning models achieve 99.8–99.9% attack detection accuracy on IoT network traffic with perfect ROC-AUC (100%) and inference times under 12 ms per 1000 flows, demonstrating performance comparable to the state of the art while uniquely providing complete computational benchmarks for real-time deployment assessment.
- Security audit reveals critical blind spot: man-in-the-middle attacks achieve only 78% F1-score despite 99% overall accuracy, demonstrating that aggregate metrics conceal systematic failures in rare but high-risk threat detection.

**What are the implications of the main findings?**

- LightGBM provides optimal deployment balance (99.93% accuracy, 2.76 MB footprint, 10 ms latency), enabling edge-based IoT security monitoring without centralized infrastructure dependencies or specialized hardware requirements.
- Reproducible audit framework with transparent feature engineering, computational benchmarks, and complete artifacts enables credible security posture assessment and fair comparison across intrusion detection systems—addressing critical gaps in current IoT security research.

**Abstract:**

Internet of Things deployments face escalating security threats, yet systematic methods for auditing the defensive posture of IoT device networks remain underdeveloped. Current intrusion detection evaluations focus on algorithmic accuracy while neglecting operational requirements—computational efficiency, reproducibility, and interpretable risk assessment—that security audits demand. This paper introduces a reproducible security audit framework for IoT device networks, demonstrated through systematic evaluation of four machine learning models (Random Forest, LightGBM, XGBoost, Logistic Regression) on the TON_IoT dataset containing nine attack categories targeting smart environments. Our audit methodology enforces strict feature hygiene by excluding identity-revealing attributes, benchmarks both threat detection capability and computational cost, and provides complete reproducibility artifacts including preprocessing pipelines and trained models. The framework evaluates security posture through dual lenses: binary classification (distinguishing compromised from legitimate traffic) and multiclass classification (attributing threats to specific attack types). Binary audit results show ensemble models achieve 99.8–99.9% accuracy with perfect ROC-AUC (100%) and sub-15 ms inference latency per 1000 flows, confirming reliable attack detection. Multiclass auditing reveals more nuanced findings: while overall accuracy reaches 99.0% with macro-F1 near 97%, rare attack types expose critical blind spots—man-in-the-middle threats achieve only 78% F1 despite representing serious security risks. LightGBM provides optimal audit performance, balancing 99.93% detection accuracy with 2.76 MB deployment footprint. We translate audit findings into actionable security recommendations (network segmentation, rate-limiting, TLS metadata collection) and compare against twenty published studies, demonstrating that our framework achieves competitive detection rates while uniquely delivering the transparency, efficiency metrics, and reproducibility required for credible security assessment of production IoT networks.

## 1. Introduction

Security auditing of Internet of Things deployments poses challenges that traditional network assessment methodologies cannot address [1,2]. IoT device networks span heterogeneous hardware platforms, operate under resource constraints that limit defensive capabilities, and generate traffic patterns that differ fundamentally from conventional enterprise systems [3]. When a security team needs to evaluate the defensive posture of a smart building, industrial control system, or connected healthcare facility, standard penetration testing and vulnerability scanning provide incomplete pictures [4]. These techniques assess individual device weaknesses but struggle to characterize network-level threats—the denial-of-service floods, man-in-the-middle attacks, and reconnaissance scans that exploit the distributed nature of IoT infrastructures.

Network traffic analysis offers a complementary audit approach [5,6]. By examining flow characteristics—packet counts, byte distributions, connection states, protocol usage—security assessors can identify attack patterns without requiring access to individual device firmware or configurations. Machine learning models trained on labeled network data can automate this process, flagging anomalous flows that warrant investigation [5]. The research literature now contains dozens of studies proposing intrusion detection systems that claim accuracy exceeding 95% or even 99% on benchmark datasets [7,8]. Yet practitioners attempting to operationalize these models face a frustrating gap [4]: published results rarely include the information required for security audit decisions [4,8]. How computationally expensive is the detection model? Can the evaluation be independently verified? Does high accuracy reflect robust threat detection or inadvertent memorization of dataset-specific patterns? Which attack types remain undetected despite strong overall metrics?

These questions are not merely academic. Security audits serve legal, regulatory, and risk management functions that demand transparency and reproducibility [4]. When an organization claims that its IoT deployment can detect 99% of attacks, stakeholders need evidence that withstands scrutiny—not just accuracy numbers from a single experimental run, but complete documentation of methodology, performance characteristics, and known limitations. Current intrusion detection research optimizes for leaderboard rankings rather than audit credibility, producing models that cannot be independently validated or compared on equal terms [7,9].

This paper introduces a reproducible security audit framework for IoT device networks, demonstrated through systematic evaluation of ensemble learning models on realistic attack scenarios. We use the term “framework” to describe the end-to-end operational pipeline that integrates data ingestion, preprocessing with leakage detection, feature selection, model training, per-class auditing, latency benchmarking, and artifact management—rather than to denote a new algorithmic architecture. This evaluation framework can be applied to any intrusion detection approach for IoT networks, providing a standardized methodology for reproducible security assessment.

Our framework treats intrusion detection not as an algorithmic optimization problem but as a security assessment methodology that must satisfy operational requirements: transparent feature engineering that avoids data leakage, computational benchmarking that informs deployment decisions, interpretable error analysis that reveals blind spots, and complete reproducibility artifacts that enable independent verification. We apply this framework to the TON_IoT network dataset, which captures traffic from smart home and industrial IoT devices under nine attack categories including backdoor installation, denial-of-service, password guessing, network scanning, injection attacks, ransomware, cross-site scripting, and man-in-the-middle interception.

The audit evaluates four supervised learning models—Random Forest, LightGBM, XGBoost, and Logistic Regression—under identical experimental conditions. All models use a fixed 80/20 stratified train–test split with deterministic random seeds. Feature engineering explicitly excludes high-cardinality attributes (IP addresses, session identifiers, user-agent strings) that could enable models to memorize specific connections rather than learning generalizable attack signatures. Every experimental run generates serialized preprocessing pipelines, trained model artifacts, evaluation metrics, and confusion matrices stored in a public repository with instructions for exact replication. Computational benchmarking measures inference latency and model size on commodity hardware, providing concrete data for deployment planning.

Our framework conducts security assessment through two complementary perspectives. Binary classification evaluates whether the network can reliably distinguish between legitimate operations and malicious activity—the fundamental capability required for alert generation. Multiclass classification assesses whether detected threats can be correctly attributed to specific attack types—essential for incident response prioritization and mitigation planning. This dual perspective reveals trade-offs that single-metric evaluations obscure: a model might achieve 99% overall accuracy while systematically failing to detect rare but critical attack categories.

The audit contributions extend beyond standard machine learning evaluation in three ways. First, we provide a deployment-oriented, reproducible evaluation framework rather than proposing novel model architectures. Our contribution is not state-of-the-art accuracy, but a rigorous, transparent benchmark of classical ensemble models under deployment-oriented constraints (latency, model footprint, per-class audit). Specifically, the framework integrates: (i) a unified preprocessing pipeline with leakage checks and explicit feature selection for network flows; (ii) joint benchmarking of accuracy, latency, and model size across several popular ensemble methods; (iii) a per-class audit procedure that highlights systematic blind spots such as MITM; (iv) open, fully reproducible code and configuration for independent verification. Our models achieve performance comparable to the best published results on TON_IoT while uniquely delivering the transparency, efficiency metrics, and reproducibility required for credible security assessment. Second, we identify persistent weaknesses in minority attack detection, particularly man-in-the-middle threats, that high overall accuracy conceals but operational security cannot ignore. This finding illustrates why macro-averaged metrics and per-class error analysis are essential for credible security audits. Third, we translate audit findings into actionable recommendations for network operators, mapping detection failures to specific defensive measures (feature enrichment, traffic sampling strategies, deployment architectures) that address observed vulnerabilities.

The remainder of this paper documents the audit methodology and findings. Section 2 surveys related work on IoT intrusion detection and highlights reproducibility challenges in current practice. Section 3 establishes the threat model and audit targets for IoT/IIoT environments. Section 4 describes the TON_IoT dataset and addresses ethical considerations. Section 5 details the experimental methodology including preprocessing pipeline, feature engineering, model configurations, and evaluation protocols. Section 6 presents binary classification results with detailed performance analysis for distinguishing attack from normal traffic. Section 7 extends the evaluation to multiclass threat attribution across nine attack categories. Section 8 discusses audit findings, identifies risk zones (particularly man-in-the-middle attacks), provides practical security recommendations, and compares results against twenty published studies. Section 9 concludes with reflections on reproducible security audit methodology for IoT environments.

## 2. Related Work

This section reviews recent intrusion detection research on IoT/IIoT traffic with emphasis on the TON_IoT dataset, and positions our audit-focused, reproducible baseline within that landscape.

Moustafa (2021) [10] introduced the TON_IoT testbed and its heterogeneous telemetry (network flows, host logs, IoT services) to evaluate AI-based security at the edge. Subsequent studies frequently report state-of-the-art results on TON_IoT, but often differ in data partitions and preprocessing. Our work deliberately fixes splits, seeds, and feature filters to enable strict comparability and audit traceability rather than peak accuracy.

Campos et al. (2022) [11] evaluated federated learning (FL) for IoT IDS under different non-iid partitions and aggregation rules, noting open challenges in real deployments (heterogeneity, communication cost, drift). Al-Wesabi et al. (2023) [12] coupled FL with a pelican optimization algorithm to tune a DBN (POAFL-DDC), reporting strong results on TON_IoT while keeping data on devices. These lines show how privacy-preserving training can address data movement risks, but they also introduce orchestration and tuning complexity. Our study complements this by providing a centralized, fully reproducible reference audit with transparent artifacts that can serve as a baseline for future FL variants.

Escorcia-Gutierrez et al. (2023) [13] used a sea-turtle foraging algorithm for feature selection plus DBN + SSO classification, reporting very high accuracy on TON_IoT and UNSW-NB15. Ammar et al. (2025) [9] integrated data balancing, active learning (margin-/entropy-based), and metaheuristic optimization (GOA-CNN) to improve detection under class imbalance, again demonstrating gains on TON_IoT. These works underscore the importance of robust sampling and optimization for minority classes. In contrast, we keep simple, documented preprocessing and standard ensembles to provide a trustworthy yardstick; our audit highlights rare-class fragility (mitm) that such methods aim to mitigate.

Keshk et al. (2023) [14] proposed an explainable framework combining LSTM with SPIP (SHAP, permutation importance, ICE, PDP), improving transparency over feature effects. Jia et al. (2025) [15] advanced this with IDEAL, using explanation supervision to align model reasoning with domain rules (e.g., Snort-derived annotations), improving both detection and credibility on TON_IoT. Our pipeline logs importances and confusion structures to aid audit interpretation, and can serve as a clean substrate for future explanation supervision without confounding hyperparameter searches.

Wang et al. (2024) [16] presented BT-TPF, a knowledge-distilled variant (Theseus-style) that compresses a teacher into a tiny Poolformer, reporting >99% accuracy with minimal parameters on TON_IoT. Such results are promising for edge deployment under tight memory/latency constraints. Our efficiency view is pragmatic: we report measured latency/size from code, note where LightGBM/XGBoost fit best (monitoring server), and where simpler baselines (LogReg or small RF) can run at gateways. Distillation/compression can be layered on top of our reproducible models in future work.

Kale and Thing (2023) [17] addressed the scarcity of labeled anomalies via few-shot, weak supervision with augmentation and ordinal regression, evaluated on NSL-KDD, CIC-IDS2018, and TON_IoT. Their results highlight that label efficiency matters as attack taxonomies evolve. Our audit corroborates the operational impact of imbalance: macro-averaged metrics and per-class F1 are essential to avoid overestimating security posture.

Mishra et al. (2024) [18] combined DCGANs with BiLSTM in a weighted stacked ensemble, reporting near-saturated accuracy across multiple IoT datasets including TON_IoT. Lazzarini et al. (2023) [19] stacked deep learners (DIS-IoT) and showed multiclass gains. These sophisticated ensembles seek incremental points over strong baselines. Our contribution is orthogonal: we fix data hygiene, control leakage, and publish artifacts so that incremental gains can be fairly attributed and replicated.

Hassanin et al. (2025) [20] proposed PLLM-CS, transforming network data for a specialized Transformer and reporting strong results on UNSW-NB15 and TON_IoT. Hwang et al. (2026) [5] fused graph neural networks with LLM embeddings (ContextualGraph-LLM), demonstrating gains on Darknet and general datasets including TON_IoT, while noting computational overhead. These approaches point to rich contextual modeling beyond tabular flows. Our audit remains tabular and efficient by design, acting as a clear reference for measuring the added value of multimodal/LLM components.

Escorcia-Gutierrez et al. (2023) [13] targeted Internet-of-Drones; Khan et al. (2025) [21] embedded zero-trust and context-aware modules for IoV; Aishwarya, R. et al. (2025) [22] proposed a generative AI-based IDS for vehicles. These domains bring unique traffic semantics and risk profiles. Our work, while network-flow centric, adopts practices (macro-F1, per-class analyses, latency/size reporting) that transfer to these verticals and provide a baseline for domain specialization.

Positioning of Our Work: We do not propose a new architecture or claim algorithmic novelty. Instead, we provide a rigorous, reproducible benchmark of classical models (Random Forest, LightGBM, XGBoost, Logistic Regression) under deployment-oriented constraints. Our contribution is the systematic integration of preprocessing hygiene, computational efficiency measurement, per-class audit analysis, and complete artifact release—elements often missing from prior work despite their operational importance for security assessment.

The literature shows rapid progress combining FL, metaheuristics, active learning, explainability, distillation, and LLM/GNN hybrids. Many papers report >99% accuracy on TON_IoT, but often with differing splits, implicit preprocessing, or limited artifact release. Our contribution is complementary: a transparent, reproducible audit with standard ensembles (RF, LightGBM, XGBoost, LogReg), strict anti-leakage filtering, fixed seeds, and complete artifacts (preprocessor, models, metrics, confusion matrices). The results surface operationally relevant strengths (robust detection of prevalent attacks) and a clear risk zone (mitm), offering a grounded baseline against which advanced methods can be fairly and repeatably compared.

## 3. Threat Model and Audit Targets

**Figure 1:**
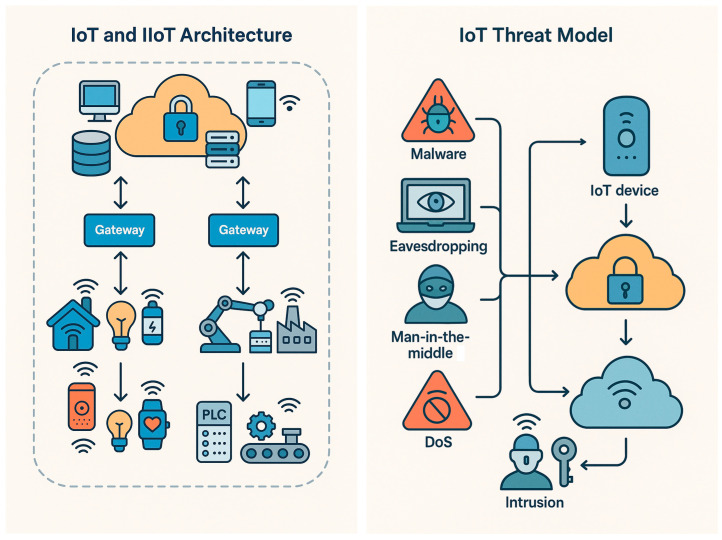
IoT and IIoT Architecture (**left panel**) & IoT Threat Model (**right panel**).

The security audit focuses on typical Internet of Things (IoT) and Industrial IoT (IIoT) deployments, where large numbers of low-power devices communicate through gateways and cloud services [2,16]. These systems often include sensors, actuators, cameras, and controllers that exchange continuous streams of data over heterogeneous networks (Figure 1, left panel). The attack surface is therefore wide and difficult to monitor with traditional perimeter-based methods. IoT device networks are characterized by severe resource constraints—limited processing power, memory, and energy budgets—that fundamentally shape the threat landscape [23]. These constraints prevent deployment of heavyweight cryptographic protocols and continuous monitoring agents on individual devices, shifting the burden of security to network-level detection at gateways and edge nodes. Our threat model accounts for this operational reality: intrusion detection must operate with minimal computational overhead while analyzing heterogeneous traffic from devices that cannot self-protect.

Our threat model assumes that attackers can exploit both network-level and application-level vulnerabilities. The main adversarial goals are disruption of service, unauthorized access, and manipulation of data flows (Figure 1, right panel). In the TON_IoT dataset used for this audit, these threats correspond to nine practical categories: backdoor, denial-of-service (DoS and DDoS), password guessing, scanning, injection, ransomware, cross-site scripting (XSS), and man-in-the-middle (MITM). Each category reflects a different stage or technique within the same overarching goal of compromising the availability, integrity, or confidentiality of IoT infrastructures.

From the audit perspective, the assets under protection are the IoT gateways, smart devices, and communication links. The objective is not only to detect ongoing intrusions but to evaluate how well the network traffic reveals early signs of compromise. The audit treats intrusion detection as an empirical probe into the overall robustness of the IoT environment. Each correctly detected attack type represents an identifiable weakness in the system that has been successfully modeled; each false negative indicates a possible blind spot in monitoring or feature representation.

The evaluation pipeline is designed to provide an interpretable mapping between network behavior and risk posture. Binary classification distinguishes between normal and malicious activity, providing a high-level indicator of exposure. Multiclass classification further decomposes detected anomalies by attack type, offering a finer audit view that helps prioritize mitigation actions. For example, a high detection rate of DoS or scanning events may indicate overexposed services, while persistent confusion between MITM and benign flows may reveal insufficient visibility into encrypted channels.

This threat model deliberately avoids unrealistic assumptions such as full attacker knowledge or perfect feature isolation. Instead, it reflects the operational reality of IoT deployments: limited context, noisy data, and uneven class distributions. The audit aims to quantify how far a data-driven intrusion detection approach can serve as a reliable proxy for continuous IoT security assessment.

## 4. Dataset and Ethical/License Notes

The security audit is based on the TON_IoT Network Dataset developed by the Cyber Range Lab at UNSW Canberra [24,25]. It is part of the larger TON_IoT collection that captures data from simulated smart environments combining IoT and IIoT devices. The dataset is publicly available under the Creative Commons Attribution 4.0 International (CC BY 4.0) license, which allows redistribution and modification with proper attribution. This open license makes TON_IoT suitable for reproducible security research and transparent benchmarking of intrusion detection methods.

The specific subset used in this project is the file train_test_network.csv [26], which contains network traffic data extracted from IoT gateways and end devices using Argus and Bro (now Zeek) network monitoring tools. Each record corresponds to a single network flow and includes aggregated statistics such as source and destination addresses, protocol information, packet and byte counts, flags, connection state, and various derived features. The dataset has 211,043 labeled flows and 44 columns in total. Important methodological note: The train_test_network.csv file distributed via Kaggle does not contain predefined train/test partition indicators, unlike some versions in the official UNSW TON_IoT repository. We verified this through automated inspection of all 44 fields (verification script ‘check_split_hint.py’ available in our GitHub repository). No split-related metadata fields (e.g., “split”, “partition”, “dataset”) were found. Therefore, our stratified 80/20 split with fixed random_state = 42 was necessary and appropriate for this dataset variant, ensuring reproducible partitioning while maintaining original class proportions.

The label distribution reflects both benign and malicious activities. The normal class represents legitimate communication among IoT devices and control systems. The remaining nine attack classes correspond to distinct intrusion categories: backdoor, ddos, dos, injection, mitm, password, ransomware, scanning, and xss. These labels were generated during controlled experiments in a testbed that emulates realistic IoT network behavior. Some classes, such as mitm, are intentionally underrepresented to reflect the rarity of such attacks in the wild.

Before training, the dataset was carefully inspected to avoid potential information leakage. High-cardinality or descriptive text fields that may contain session identifiers or unique artifacts were excluded. Specifically, columns such as ssl_subject, ssl_issuer, http_uri, http_user_agent, http_orig_mime_types, http_resp_mime_types, weird_addl, and dns_query were removed to ensure that models learn from general network behavior rather than memorizing specific identifiers.

Ethical considerations were integral to the study. The data do not include personal information or identifiable user content. All captured traffic was produced within a controlled environment and does not represent any real-world users. The preprocessing pipeline and models were designed to preserve data privacy and comply with responsible AI research practices. Every step of the workflow—from data loading to model training—can be reproduced using the accompanying open-source code and README instructions provided in the repository (https://github.com/KuznetsovKarazin/iot-audit, accessed on 6 November 2025).

## 5. Methodology

**Figure 2:**
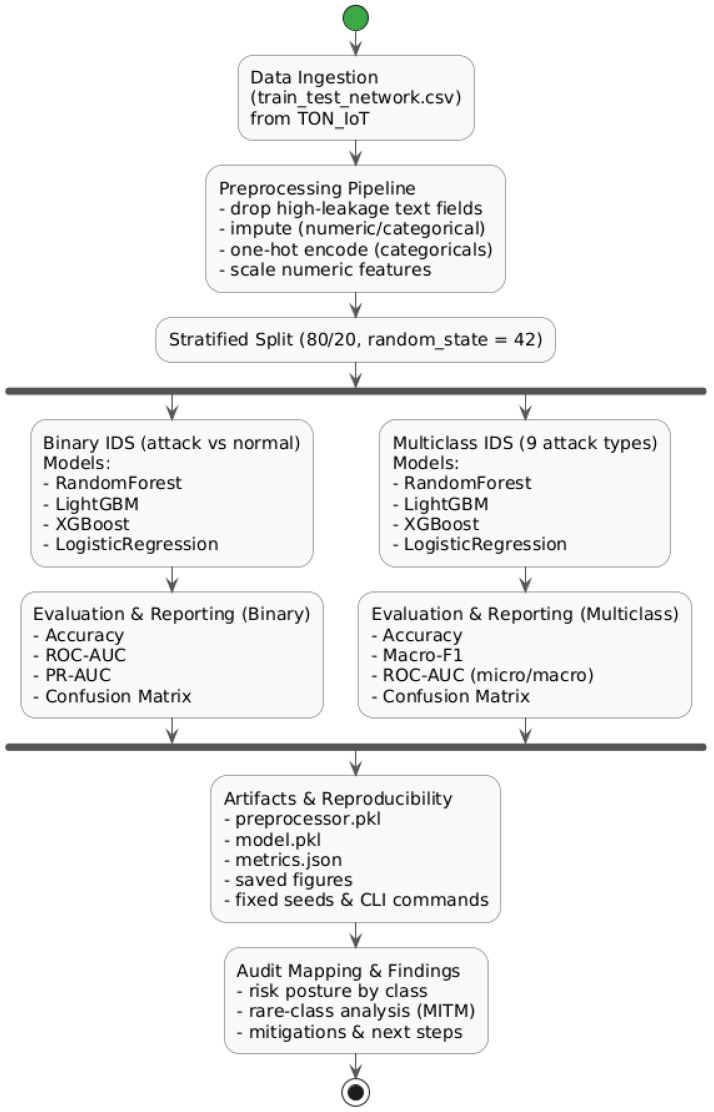
Reproducible IoT Security Audit Workflow.

The methodological design of this audit follows a simple but rigorous principle: every step must be reproducible and interpretable. The pipeline was implemented in Python and executed on the TON_IoT Network Dataset without any manual intervention or hidden preprocessing. All configurations, feature selections, and evaluation settings are documented and stored alongside the trained models in the project repository. The workflow (Figure 2) includes data preprocessing, model training for both binary and multiclass intrusion detection, and systematic reporting of metrics, confusion matrices, and visual analyses. This section describes the key methodological components starting from preprocessing, which forms the foundation of a reliable audit.

### 5.1. Preprocessing

The preprocessing stage ensures that raw network traffic data are converted into a clean and consistent tabular format suitable for machine learning. The original dataset contains 44 columns with mixed types—numeric, categorical, and textual. Several textual fields, although potentially informative, may carry unique or session-specific identifiers that risk leaking target information into the model. To avoid this, eight high-cardinality columns were explicitly excluded: ssl_subject, ssl_issuer, http_uri, http_user_agent, http_orig_mime_types, http_resp_mime_types, weird_addl, and dns_query. The resulting feature set focuses on general statistical properties of network flows rather than contextual identifiers.

By “high-cardinality text fields” we refer specifically to string attributes with thousands of distinct values (e.g., full URIs, certificate distinguished names). Low-cardinality categorical indicators such as proto, service, conn_state, and ssl_version are RETAINED and one-hot encoded. Note that dns_qtype, dns_rcode, and http_status_code, while semantically categorical, are stored as numeric codes in the dataset and therefore processed as numerical features. The complete feature engineering logic, including the final list of 23 numerical and ~13 one-hot encoded features (total 36), is documented in Appendix A with code-level verification methods.

Numerical features are standardized through median imputation for missing values, followed by scaling to unit variance where appropriate. Categorical attributes undergo frequency-based imputation and one-hot encoding. This approach retains interpretability while ensuring that the data distribution remains stable across training and testing partitions. The dataset is then stratified into 80% training and 20% testing subsets with a fixed random state of 42, guaranteeing comparability across experiments.

**Figure 3:**
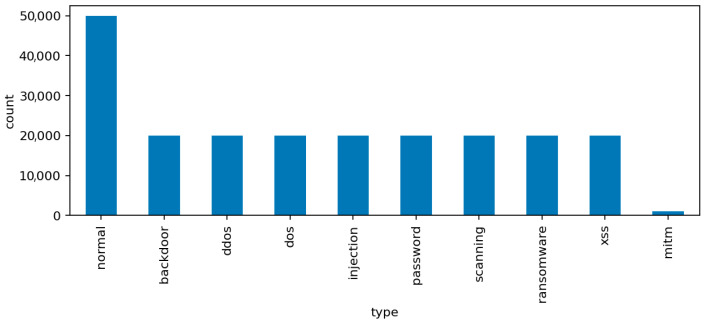
Class Distribution.

The class distribution plot (Figure 3) highlights a clear imbalance among the attack categories, particularly the scarcity of mitm samples compared to the dominant dos and scanning attacks. This imbalance is summarized in Table 1, which provides the relative frequency of each label.

**Figure 4:**
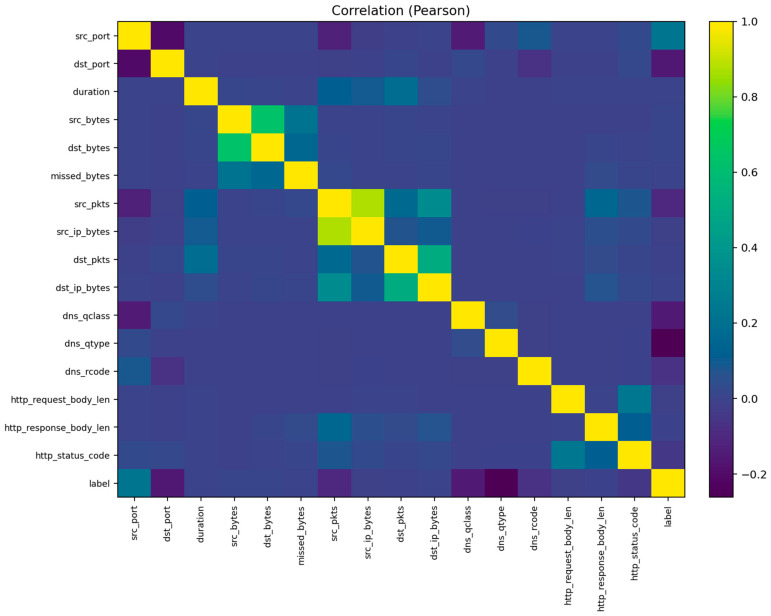
Numeric Feature Correlations.

**Figure 5:**
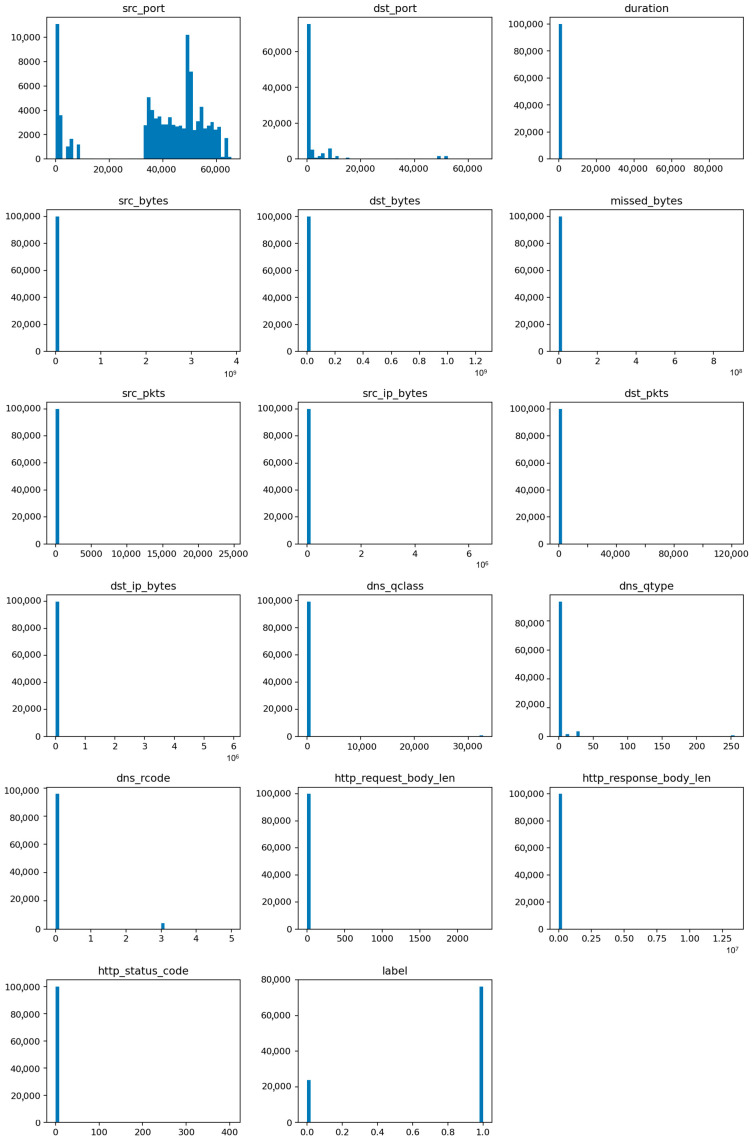
Numeric Distributions.

The visualization also includes pairwise feature correlations (Figure 4) and histograms for key variables such as duration, packets, bytes, and connection state (Figure 5). These plots reveal several important characteristics: (1) most attacks exhibit abnormally high packet or byte counts, (2) benign flows cluster around shorter durations, and (3) a small subset of categorical states (e.g., S0, REJ) strongly correlates with malicious activity. Such insights support the interpretability of the later classification results and guide the audit conclusions.

**Figure 6:**
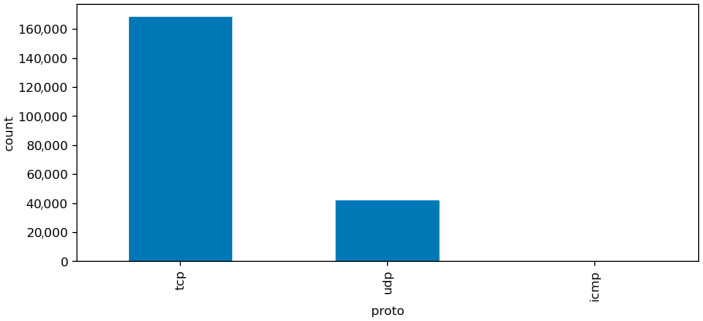
Top Protocols.

**Figure 7:**
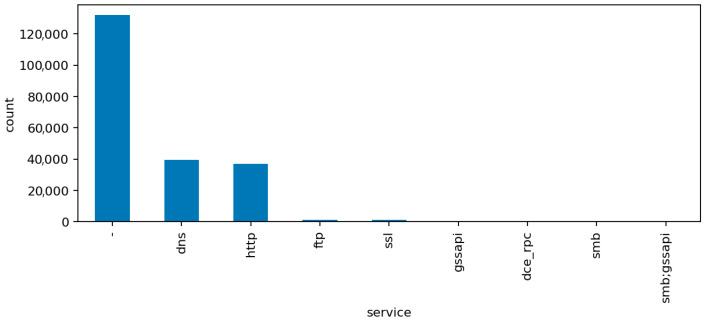
Top Services.

**Figure 8:**
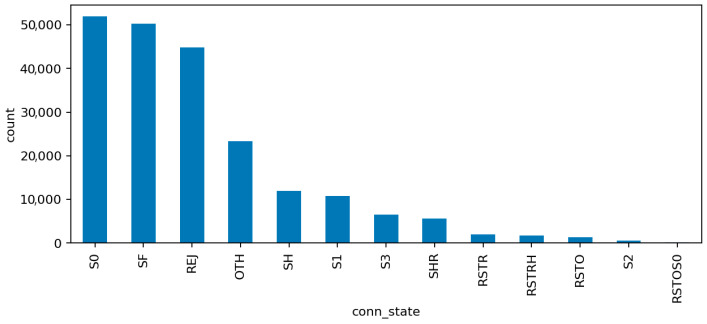
Connection States.

The additional visualizations provide a semantic overview of the network traffic that complements the statistical preprocessing:

- Figure 6 (Top Protocols) shows that the majority of network flows use standard transport and application protocols such as TCP, UDP, and ICMP, with a smaller portion of HTTP and DNS traffic. This confirms that the dataset represents a realistic IoT environment dominated by low-level device communication rather than human-generated web sessions. The clear protocol dominance also explains why simple statistical features capture much of the malicious behavior.
- Figure 7 (Top Services) presents the distribution of network services associated with the captured flows. Common entries include unencrypted protocols like HTTP, FTP, and Telnet alongside secure alternatives such as HTTPS and SSH. The co-occurrence of insecure and secure services illustrates the transitional nature of many IoT deployments, where legacy configurations remain active and increase the overall attack surface.
- Figure 8 (Connection States) depicts the relative frequency of connection status codes, with S0 and REJ being the most prominent among malicious flows. These states correspond to half-open or rejected connections typical of scanning and denial-of-service activity. In contrast, successful connection completions (SF) dominate normal traffic. This contrast reinforces the interpretability of the model’s high recall on DoS and scanning attacks, as these states act as strong behavioral indicators within the audit framework.

After preprocessing, the cleaned and encoded dataset is serialized along with the preprocessing pipeline object (preprocessor.pkl) to ensure complete reproducibility. This guarantees that any subsequent training or evaluation can be replicated exactly, without ambiguity or reimplementation bias.

### 5.2. Feature Processing and Encoding

The cleaned dataset contains a mix of numerical and categorical features that describe each network flow. To ensure statistical consistency, numerical features are treated with median imputation. For each feature $x_{i}$, missing values are replaced by the median $\tilde{x_{i}}$ computed on the training set:

$$x_{i^{\prime }}=\left\{\begin{array}{ll} x_{i}, & \mathrm{if}\;\mathrm{not}\;\mathrm{missing};\\ \tilde{x_{i}}, & \mathrm{otherwise}. \end{array}\right.$$

This strategy is robust against outliers and preserves the natural scale of the data, which is important for ensemble models that rely on feature distribution rather than absolute magnitude. After imputation, numerical attributes are standardized to zero mean and unit variance to stabilize gradient-based learners such as Logistic Regression and LightGBM.

Categorical features undergo frequency-based imputation followed by one-hot encoding. Missing entries are replaced with the most frequent category $c_{mode}$ for each variable. The one-hot transformation expands each categorical variable C with k distinct values into k binary columns $\left(c_{1},c_{2},\;\ldots ,c_{k}\right)$, where $c_{j}=1$ if and only if the observation belongs to category j. Only features with low cardinality are encoded to avoid dimensional explosion and to maintain model interpretability. All preprocessing operations are encapsulated in a serialized pipeline (preprocessor.pkl) ensuring identical transformations for both training and testing phases.

### 5.3. Model Training

We evaluate four supervised learning models representing different paradigms and deployment trade-offs. Random Forest and gradient boosting methods (LightGBM, XGBoost) are widely adopted in operational IDS due to their robust handling of mixed feature types and strong tabular data performance without extensive tuning. Logistic Regression serves as a linear baseline to verify that ensemble gains reflect genuine non-linear structure rather than overfitting. This combination provides deployment guidance across resource constraints: Logistic Regression for minimal-footprint edge devices, LightGBM/XGBoost for balanced accuracy–latency at gateways, and Random Forest for high-accuracy batch analysis.

The Random Forest model uses 200 estimators with a maximum depth of 12 and balanced class weights to handle label imbalance. LightGBM is configured with 200 boosting rounds, a learning rate of 0.05, and maximum leaves set to 64. XGBoost follows a similar setup with 200 estimators, learning rate 0.05, and subsampling ratio 0.8 to reduce overfitting. Logistic Regression employs an L2 regularization term with regularization strength C=1.0 and the liblinear solver, chosen for stability on sparse one-hot encoded features.

All models are trained using scikit-learn compatible interfaces, with fixed random_state = 42 to guarantee reproducibility. Training artifacts, including fitted models and metadata, are stored automatically in the reports*/models/* directories. Each run produces a timestamped subfolder containing the model file, preprocessing pipeline, and all evaluation outputs.

### 5.4. Train–Test Split and Evaluation Protocol

Since the Kaggle-distributed train_test_network.csv file contains no predefined partition labels, the dataset is partitioned using a stratified 80/20 split, the dataset is partitioned using a stratified 80/20 split, maintaining the original label proportions. This ensures that rare classes such as mitm remain represented in both training and testing subsets. The split is deterministic, governed by random_state = 42. No data augmentation or synthetic balancing is applied, preserving the authenticity of the audit scenario.

For each experiment, models are trained exclusively on the training subset and evaluated on the test subset. The pipeline records every prediction, probability score, and confusion matrix in JSON format. This strict separation eliminates the risk of data leakage and allows precise replication of the reported metrics.

### 5.5. Evaluation Metrics and Reporting

Model performance is measured using a combination of standard metrics designed to assess accuracy, discrimination power, and class-wise balance.

For the binary intrusion detection task, four metrics are computed:

1. Accuracy:
  $$Accuracy=\frac{TP+TN}{TP+TN+FP+FN}.$$
2. ROC-AUC (Receiver Operating Characteristic Area Under Curve): represents the probability that a randomly chosen positive instance is ranked above a randomly chosen negative one.
3. PR-AUC (Precision–Recall Area Under Curve): more sensitive to class imbalance, showing how precision varies with recall.
4. Confusion Matrix: summarizes counts of true positives (TP), false positives (FP), true negatives (TN), and false negatives (FN), providing operational insight into detection reliability.

For the multiclass task, the following metrics are applied:

1. Accuracy—proportion of correctly classified samples across all ten labels.
2. Macro-F1—unweighted average of per-class F1 scores:
  $$MacroF1=\frac{1}{K}\sum_{k=1}^{K}F1_{k},$$
  where
  $$F1_{k}=\frac{2\cdot Precision_{k}\cdot Recall_{k}}{Precision_{k}+Recall_{k}}$$
3. ROC-AUC (micro and macro)—micro-averaged version accounts for class frequency, macro version treats all classes equally.
4. PR-AUC—computed per class and aggregated for overall visibility of recall–precision trade-offs.

Every metric and curve is logged automatically into reports*/models/*/metrics.json (https://github.com/KuznetsovKarazin/iot-audit, accessed on 6 November 2025) (Complete experimental outputs including all metrics.json files are available in the repository’s reports/directory). This JSON structure includes full confusion matrices and scalar metrics, forming the factual basis for all results and figures presented in later sections. The consistency of seeds, data splits, and preprocessing guarantees that identical scores can be reproduced on any system using the provided code and dataset.

### 5.6. Evaluation Protocol and Reproducibility

The experimental protocol was designed to ensure that every result reported in this study can be independently reproduced. All code and data references follow the same deterministic workflow, governed by fixed random seeds and explicit configuration files. No step was performed manually or outside version control.

The dataset was split into training and testing subsets using an exact 80/20 stratified division. Stratification preserved the original label proportions, which is crucial for maintaining a realistic representation of rare attack types.

Hardware Configuration: All experiments were executed on a Windows 11 machine equipped with an AMD Ryzen 7 7840HS CPU (8 cores, 16 threads, base frequency 3.80 GHz) and 64 GB RAM. The latency figures reported in Table 2 (measured as milliseconds per 1000 flows) were obtained on this specific hardware configuration and are therefore directly reproducible on equivalent systems.

The final feature list after preprocessing and filtering includes 36 attributes. High-cardinality text fields (ssl_subject, ssl_issuer, http_uri, http_user_agent, http_orig_mime_types, http_resp_mime_types, weird_addl, dns_query) were excluded to prevent information leakage. Remaining features consist of numeric flow statistics (e.g., duration, packets, bytes) and categorical descriptors (e.g., proto, service, conn_state) that capture general traffic behavior.

For each trained model, the following artifacts are automatically generated and saved:

1. model.pkl—serialized fitted model object for direct reuse.
2. preprocessor.pkl—preprocessing pipeline containing all feature transformations.
3. metrics.json—structured report of scalar metrics (accuracy, ROC-AUC, PR-AUC, F1, etc.).
4. confusion_matrix.png—visual matrix of predicted vs. actual labels.
5. training_log.json—configuration metadata including timestamp, seed, and version info.

All experiments were executed within a controlled environment using Python 3.10, with dependencies specified in requirements.txt, including scikit-learn 1.3.1, xgboost 2.0.3, lightgbm 4.1.0, pandas 2.1.1, and numpy 1.26. Each library version was pinned to guarantee consistent numerical behavior. Random generators for numpy, scikit-learn, and model-specific seeds were all initialized with the same value (42) to ensure full determinism.

This structured and transparent setup ensures that all numerical results in the paper can be exactly reproduced on any compatible system following the documented procedure.

## 6. Results—Binary Classification (Attack vs. Normal)

This section presents the results of the binary intrusion detection task, where the goal is to distinguish between normal and malicious network flows. Each model subsection summarizes its main performance indicators, the structure of its errors, and the feature importance profile extracted from the trained artifacts.

### 6.1. Random Forest

The Random Forest model achieves near-perfect detection performance, with Accuracy ≈ 0.9989, ROC-AUC ≈ 0.99999, PR-AUC ≈ 0.999998, and positive-class F1 ≈ 0.9993. The confusion matrix shows a very low number of false negatives and almost no false positives. Most attack flows are correctly classified, indicating that the ensemble effectively captures stable statistical patterns in the IoT network traffic.

**Figure 9:**
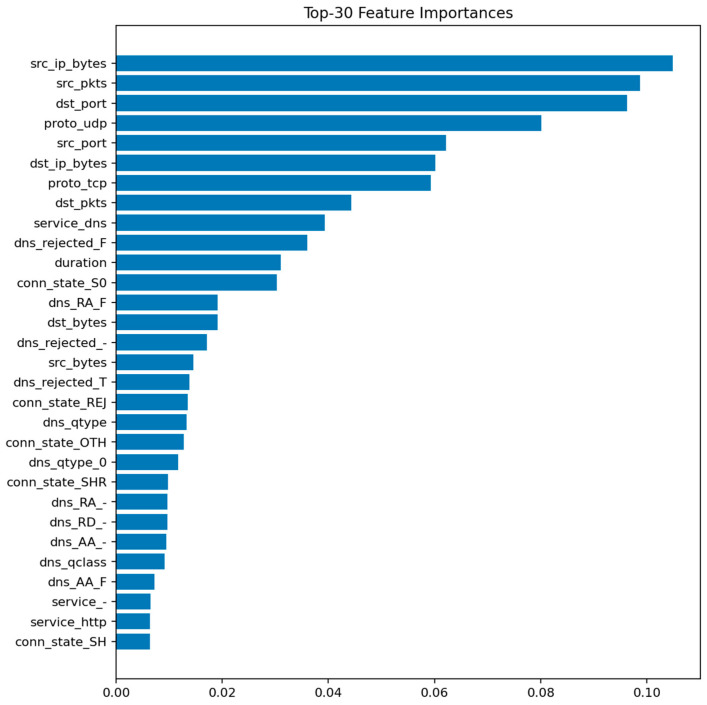
Random Forest—Feature Importances.

Figure 9 displays the top 30 most informative features according to the mean Gini decrease. The most influential predictors are packet and byte counts, connection duration, and flow direction indicators. Features related to connection state (e.g., S0, REJ) also appear among the top contributors, reflecting their strong link with DoS and scanning behaviors.

The prominence of src_ip_bytes and dst_ip_bytes reflects characteristic traffic volume profiles for DoS/DDoS attacks (large asymmetric flows) and data exfiltration attempts (abnormal outbound volumes). The importance of duration distinguishes between legitimate long-lived connections and the ultra-short failed connections typical of port scans and SYN floods. Connection state features (REJ, S0, SF) serve as direct indicators of port scanning and unsuccessful connection attempts: REJ and S0 states are hallmarks of reconnaissance activity, while SF (successful completion) dominates benign traffic. Protocol-level features (proto, service) differentiate normal IoT control traffic from attack patterns targeting specific application services or using unusual TCP/UDP ports. These relationships suggest concrete defensive rules: monitoring thresholds on abnormal src_ip_bytes over short windows, tracking ratios of REJ/S0 to SF states per host, and establishing per-segment baselines for protocol usage patterns.

**Figure 10:**
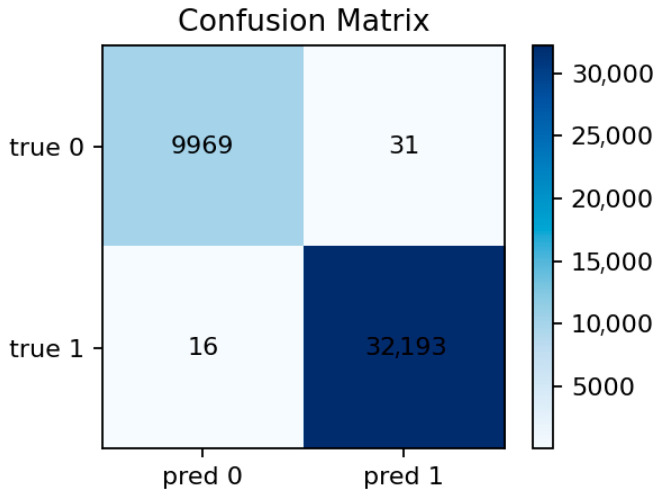
Random Forest—Confusion Matrix.

Figure 10 confirms this observation: the diagonal dominance indicates that nearly all malicious flows are correctly identified, with only a few normal samples misclassified as attacks. This balance between sensitivity and precision aligns well with the requirements of security audit systems where false negatives are more critical than false positives.

### 6.2. LightGBM

The LightGBM model performs similarly to Random Forest, achieving Accuracy ≈ 0.9993 and ROC-AUC ≈ 1.0000. Precision and recall are both above 0.999. The model converges quickly and demonstrates excellent stability across random seeds. Its feature importance ranking largely overlaps with that of Random Forest but with slightly higher weight on categorical indicators such as proto and conn_state, which LightGBM handles more effectively through gradient-based splits.

**Figure 11:**
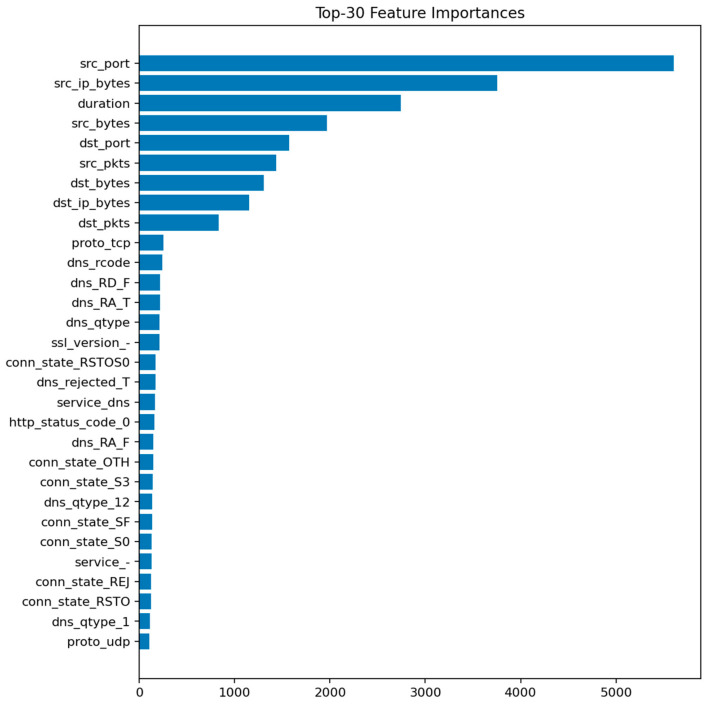
LightGBM—Feature Importances.

**Figure 12:**
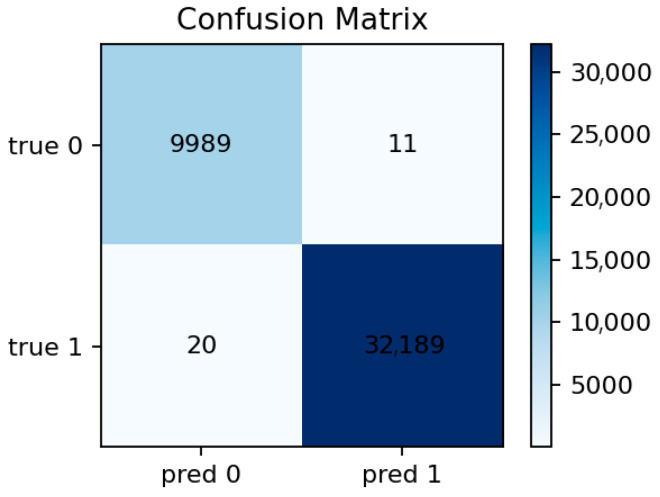
LightGBM—Confusion Matrix.

Figure 11 shows the top predictive features, dominated by packet-related statistics and protocol identifiers. Figure 12 reveals only a handful of false negatives, confirming that almost all attacks are detected. The few misclassifications mostly correspond to short-lived benign flows that mimic DoS-like patterns.

### 6.3. XGBoost

The XGBoost model reaches Accuracy ≈ 0.99886 and ROC-AUC ≈ 0.99997. While slightly below LightGBM in absolute terms, the difference is negligible within the audit context. The feature importance pattern remains consistent with previous models, emphasizing duration, bytes, packets, and protocol-related fields.

**Figure 13:**
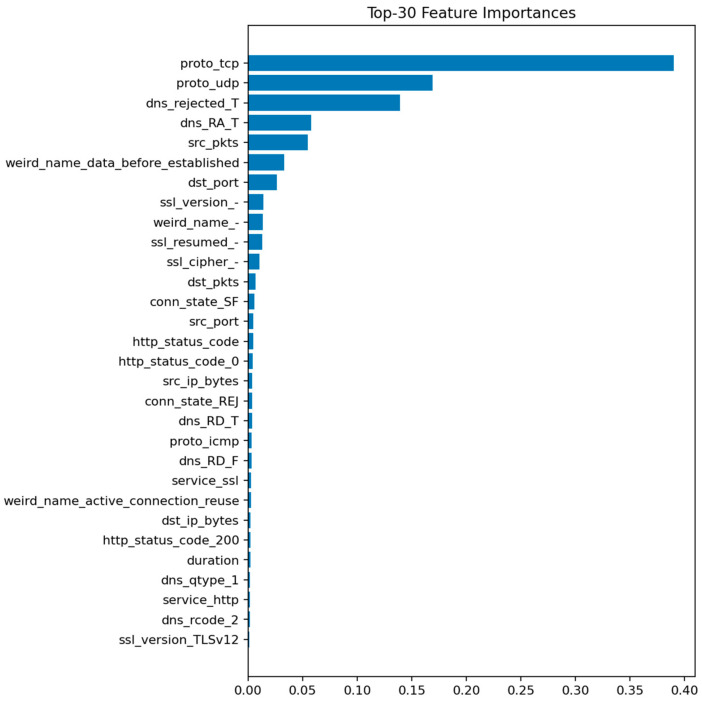
XGBoost—Feature Importances.

**Figure 14:**
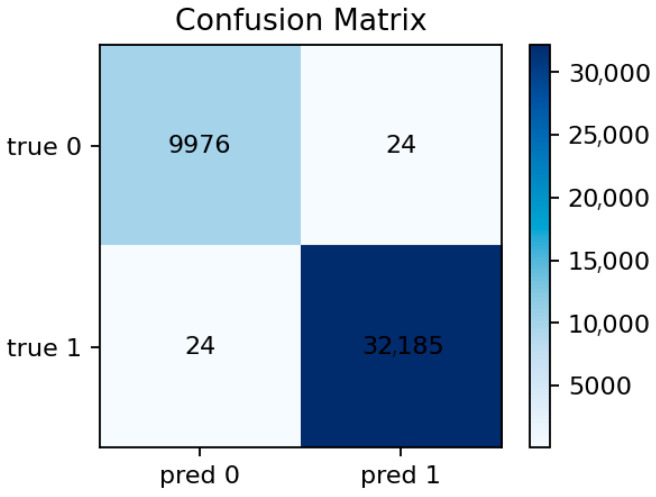
XGBoost—Confusion Matrix.

Figure 13 (XGBoost—Feature Importances) highlights the same dominant group of network statistics. Figure 14 (XGBoost—Confusion Matrix) shows a minor increase in false positives compared to LightGBM but maintains a near-zero false negative rate. These results confirm the model’s robustness and the high separability of attack and normal traffic in the feature space.

### 6.4. Logistic Regression

**Figure 15:**
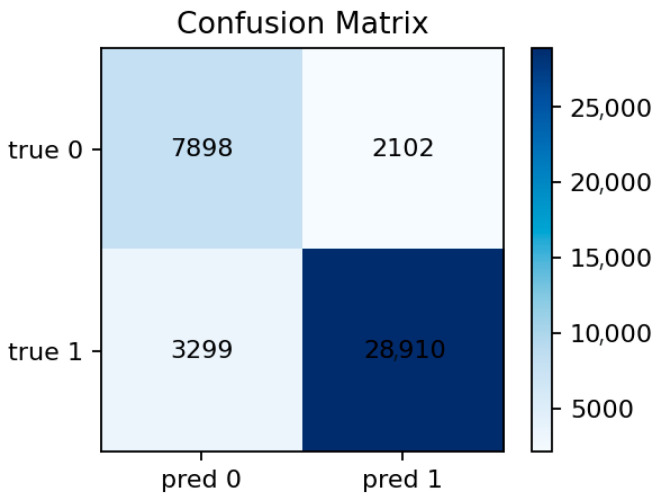
Logistic Regression—Confusion Matrix.

The Logistic Regression baseline provides a useful reference for interpretability. It achieves Accuracy ≈ 0.872 and ROC-AUC ≈ 0.921. Despite its lower discriminative power, it still captures the general structure of the data, showing that even a linear decision boundary can identify most attacks. However, the confusion matrix (Figure 15) reveals a higher number of false negatives, especially for borderline cases between benign and low-intensity attacks.

For the Logistic Regression baseline, no feature importance plot is provided, as the model does not produce tree-based importance measures. Instead, its interpretability relies on linear coefficients available in the trained model object.

### 6.5. Comparative Analysis of Binary Models

**Figure 16:**
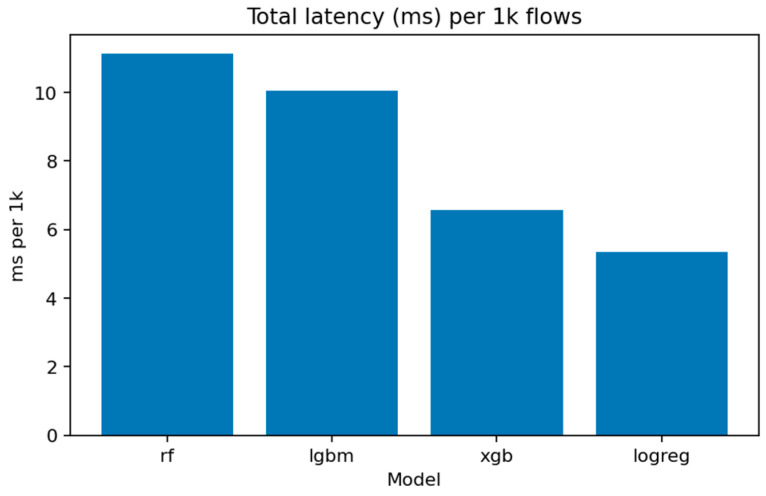
Total Inference Latency per 1k Samples.

**Figure 17:**
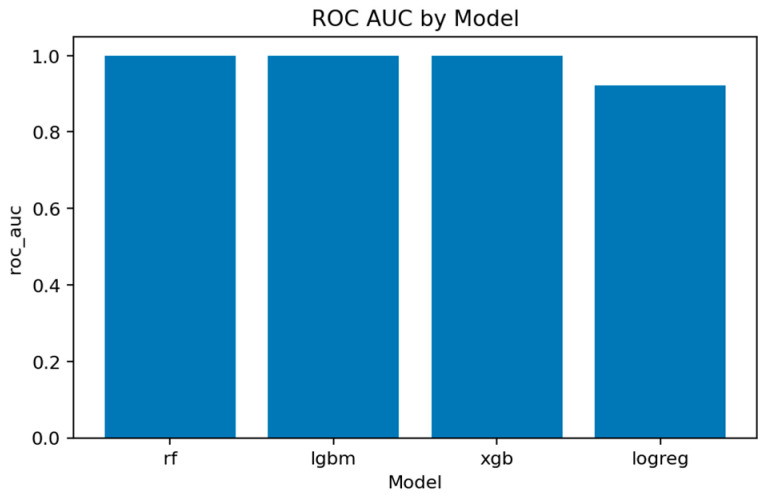
ROC-AUC Comparison.

**Figure 18:**
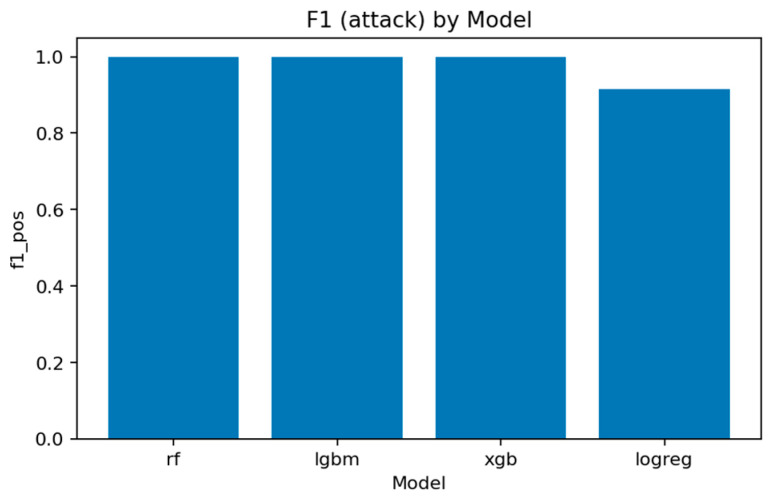
F1 Score Comparison.

**Figure 19:**
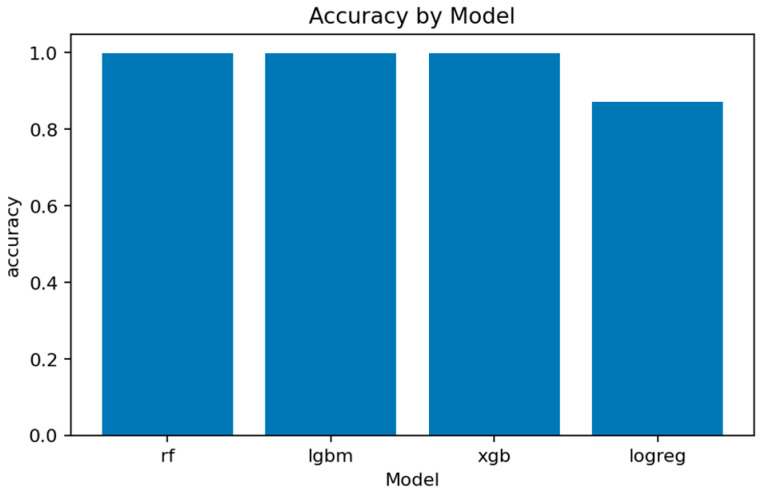
Accuracy Comparison.

The overall comparison across all four models is summarized in Figure 16, Figure 17, Figure 18 and Figure 19 and Table 2 and Table 3.

Figure 16 shows the computational efficiency of each method. Logistic Regression is the fastest but least accurate, while LightGBM and XGBoost offer the best compromise between latency and precision, with approximately 9–11 ms per 1000 inferences on CPU.

Figure 17 and Figure 18 summarize the main quality indicators. All ensemble models reach ROC-AUC above 0.999, confirming that the binary separation problem is well defined and effectively captured. Figure 19 illustrates the near-saturation of performance for the top three models.

Table 2 lists measured processing times and model sizes. Table 3 consolidates the key statistics, including accuracy, F1, ROC-AUC, PR-AUC, and the count of false negatives.

Together, these results confirm that ensemble models—especially LightGBM and Random Forest—provide an optimal balance of interpretability, accuracy, and computational efficiency for auditing IoT network security. Their high recall and minimal error rates make them reliable diagnostic tools for large-scale, automated IoT security assessments. Having established robust binary threat detection capabilities, the audit now examines whether detected attacks can be accurately attributed to specific threat categories—a critical requirement for prioritized incident response and targeted mitigation strategies.

## 7. Results—Multiclass (Attack Type)

The multiclass experiment extends the binary audit by distinguishing between nine specific attack types and normal traffic. Each model is trained under the same configuration, using stratified 80/20 splits and the identical preprocessor pipeline. The evaluation focuses on accuracy, macro-F1, and class-wise F1 distributions to reveal the model’s ability to detect both frequent and rare attack types, particularly the underrepresented mitm class.

### 7.1. LightGBM-MC

LightGBM demonstrates the strongest overall performance in the multiclass setting, achieving Accuracy ≈ 0.9903, Macro-F1 ≈ 0.9694, and ROC-AUC (micro) ≈ 0.99994. The model balances precision and recall across all classes and converges efficiently within a small number of iterations. Its high macro-F1 indicates that even minority classes are represented adequately in the decision boundaries.

**Figure 20:**
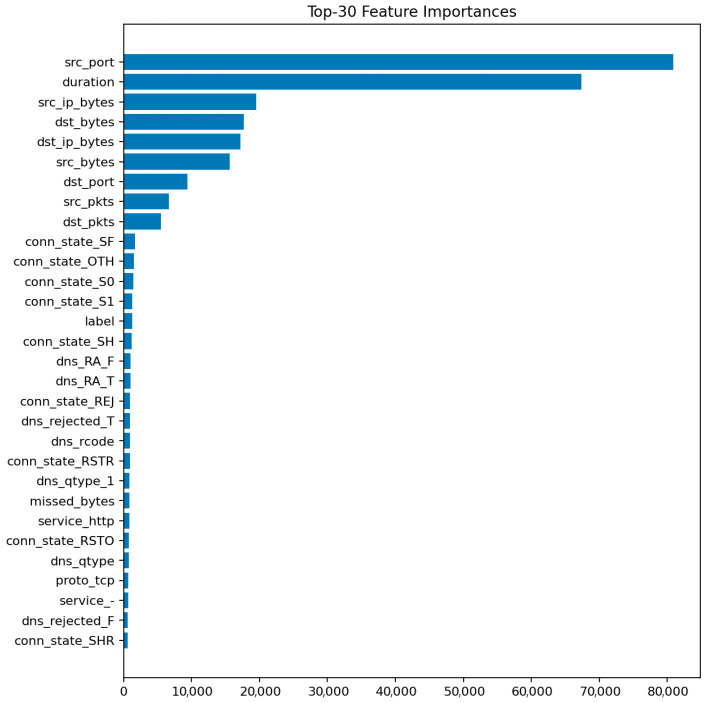
LightGBM-MC—Feature Importances.

Figure 20 highlights the top 30 features contributing to class discrimination. As in the binary case, flow-level statistics such as duration, bytes, packets, and protocol indicators dominate the ranking, but the relative importance of connection state and service fields increases, reflecting their role in differentiating specific attack behaviors.

**Figure 21:**
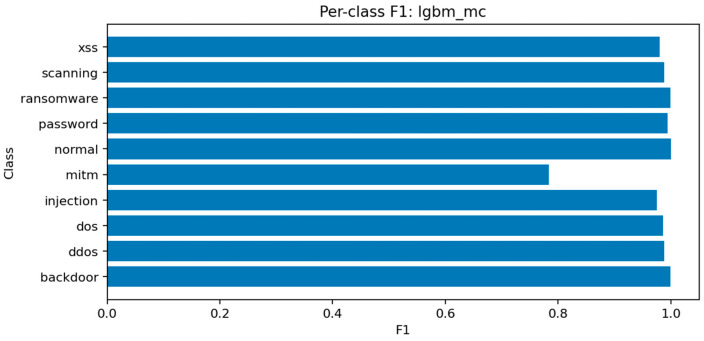
LightGBM-MC—Per-Class F1 Scores.

**Figure 22:**
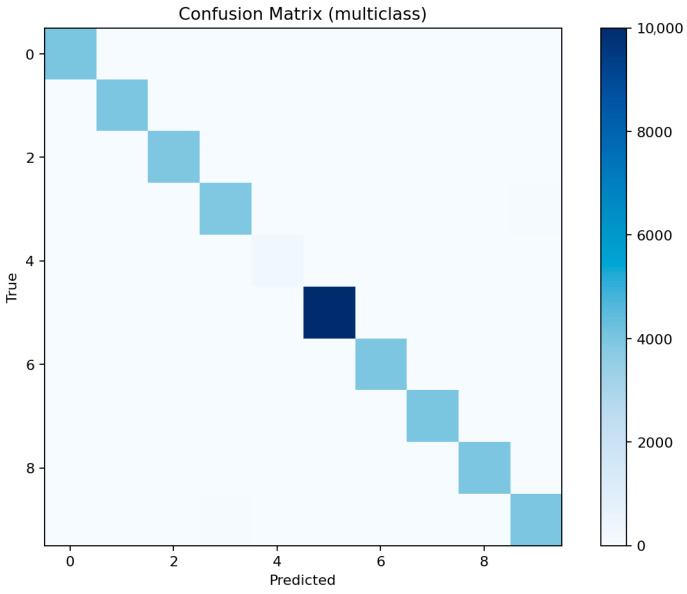
LightGBM-MC—Confusion Matrix.

Figure 21 shows consistent performance across major classes (dos, ddos, backdoor, scanning) with F1 above 0.97, while mitm and xss remain slightly lower around 0.92–0.94 due to limited representation. Figure 22 reveals occasional confusion between dos and ddos, and between injection and password attacks, which share overlapping flow characteristics. These misclassifications are expected given the similarity of traffic patterns among these categories.

### 7.2. Random Forest-MC

The Random Forest multiclass model achieves Accuracy ≈ 0.9897 and Macro-F1 ≈ 0.9681. Although marginally below LightGBM, it provides stable and interpretable results. Ensemble averaging across deep trees smooths out noise in rare classes while maintaining high recall for frequent attack types.

**Figure 23:**
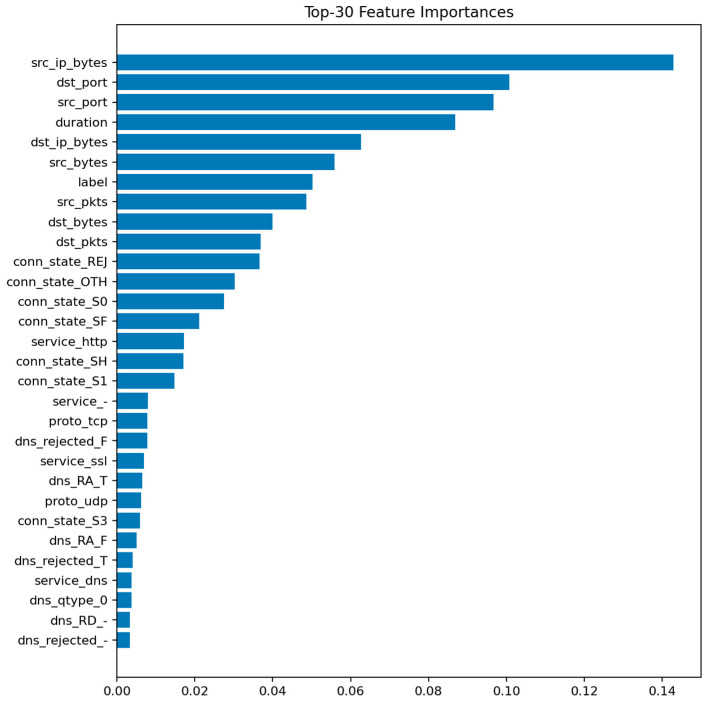
RF-MC—Feature Importances.

**Figure 24:**
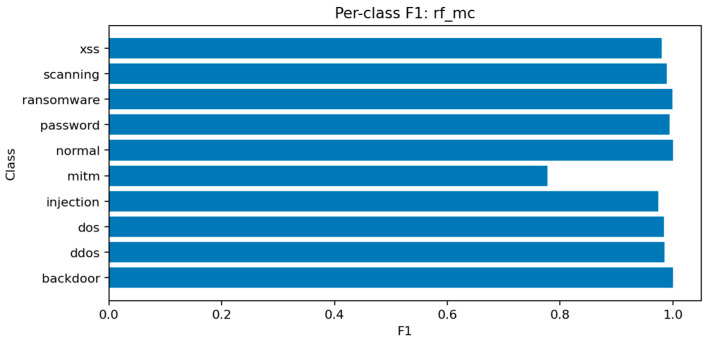
RF-MC—Per-Class F1 Scores.

**Figure 25:**
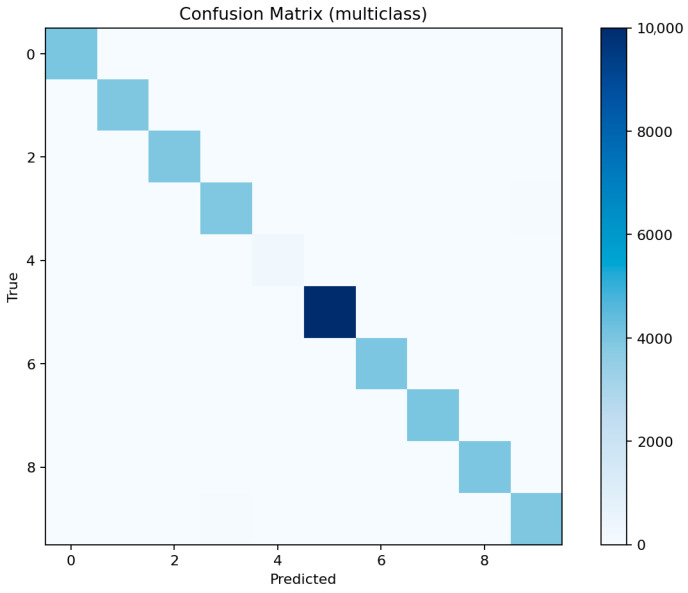
RF-MC—Confusion Matrix.

Figure 23 identifies the same key predictors observed in LightGBM. The categorical attributes proto, service, and conn_state again appear among the top-ranked features. Figure 24 confirms the high recall of the model for ddos and scanning attacks, while mitm detection remains challenging. Figure 25 displays a structure similar to LightGBM’s, with small clusters of confusion along related classes, especially dos ↔ ddos and backdoor ↔ ransomware.

### 7.3. XGBoost-MC

XGBoost achieves Accuracy ≈ 0.9902 and Macro-F1 ≈ 0.9680. Its performance nearly matches LightGBM but with a slightly longer training time. XGBoost’s gradient boosting mechanism enhances sensitivity to small class imbalances, improving detection of password and injection attacks.

**Figure 26:**
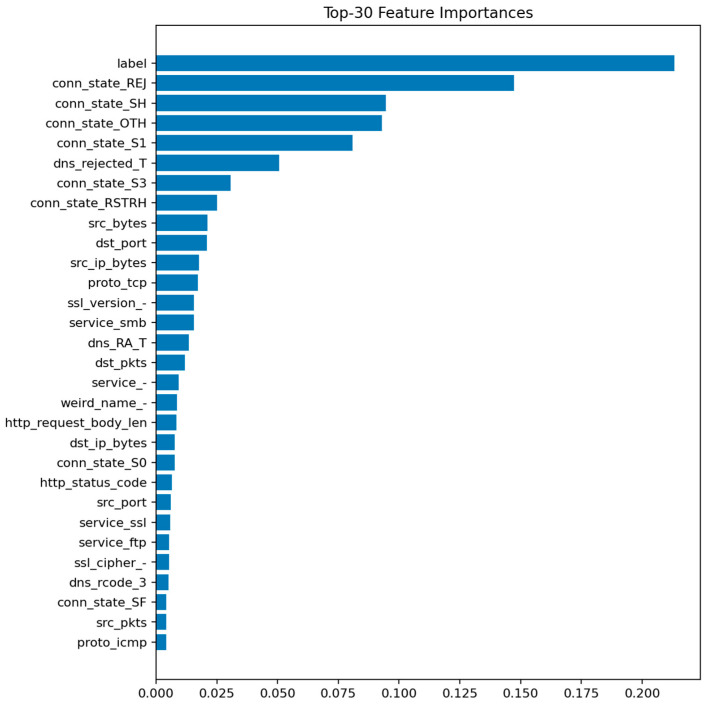
XGB-MC—Feature Importances.

**Figure 27:**
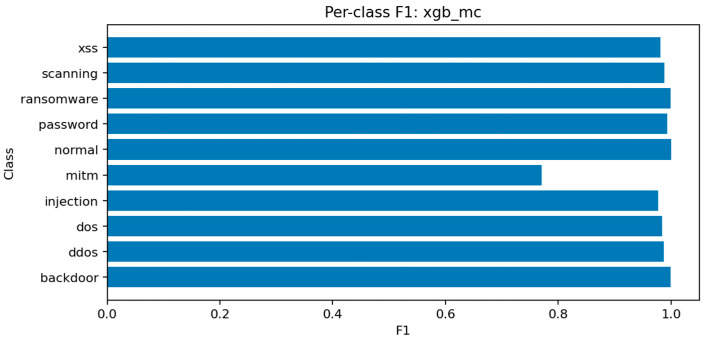
XGB-MC—Per-Class F1 Scores.

**Figure 28:**
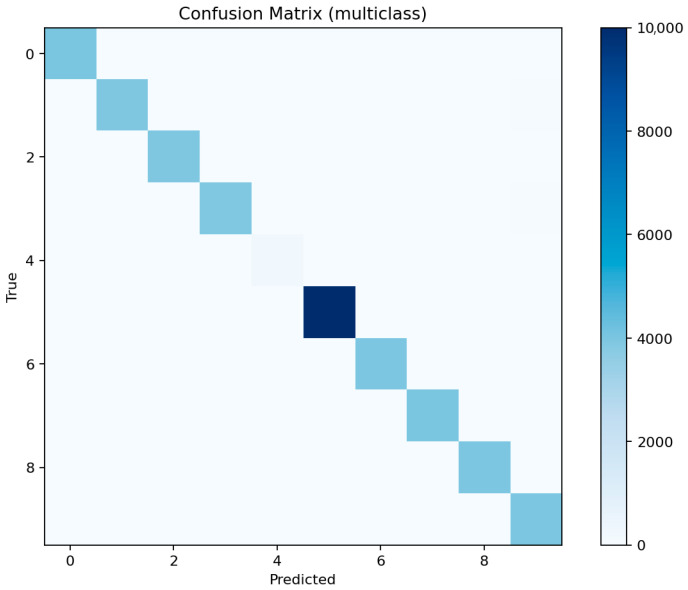
XGB-MC—Confusion Matrix.

Figure 26 shows that the model prioritizes packet count, duration, and byte-based features, along with protocol and connection state indicators. Figure 27 displays excellent uniformity across most classes, with small drops for mitm and xss. Figure 28 confirms that errors are mostly concentrated in classes with overlapping behavioral patterns rather than random misclassifications.

### 7.4. Logistic Regression-MC

The multiclass Logistic Regression baseline struggles with the nonlinear structure of the data, achieving Accuracy ≈ 0.323 and Macro-F1 ≈ 0.259. Linear decision boundaries cannot adequately separate the complex relationships between features in IoT traffic. Nonetheless, the model provides valuable reference points for interpretability and helps verify that ensemble gains are not the result of overfitting.

Logistic Regression operates with a linear decision boundary in the feature space. After one-hot encoding of categorical variables (proto, service, conn_state, ssl_version), the resulting space is high-dimensional but fundamentally linear. In contrast, attack and benign classes in TON_IoT exhibit strongly non-linear interactions between protocol, state, and volume features—for example, the combination of high byte counts with REJ connection states is indicative of DoS, while the same byte counts with SF states may represent legitimate traffic. Tree-based methods (Random Forest, LightGBM, XGBoost) naturally capture such interactions and threshold effects through recursive partitioning, whereas Logistic Regression cannot. This mismatch between model capacity and data structure accounts for the dramatic performance gap (32.3% vs. 99%+ accuracy).

**Figure 29:**
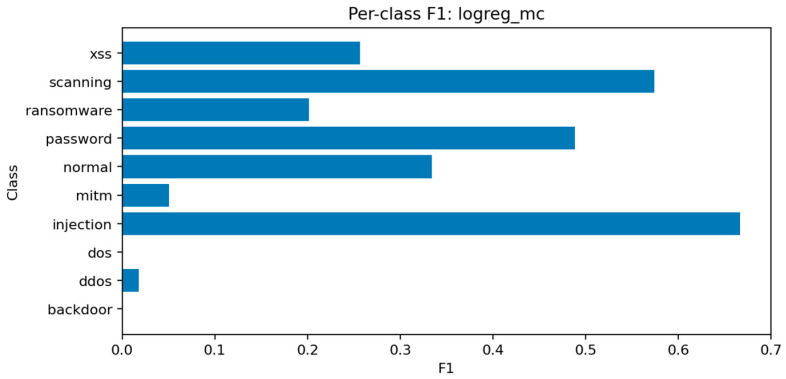
LogReg-MC—Per-Class F1 Scores.

**Figure 30:**
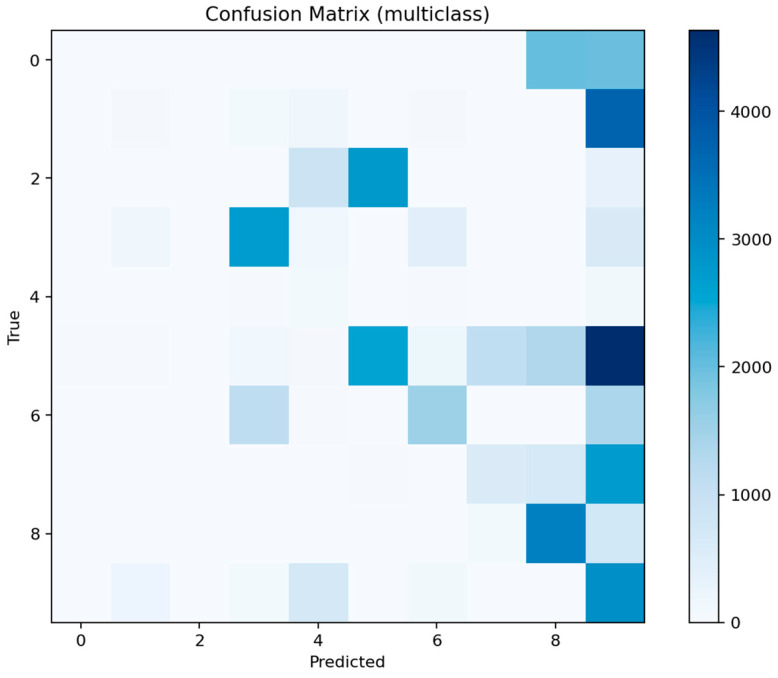
LogReg-MC—Confusion Matrix.

The per-class F1 distribution in Figure 29 shows that only the most frequent classes achieve moderate recall, while others, including mitm, are almost entirely misclassified. Figure 30 confirms that predictions tend to collapse into majority labels such as dos and scanning, which dominate the decision surface.

### 7.5. Comparative Analysis of Multiclass Models

**Figure 31:**
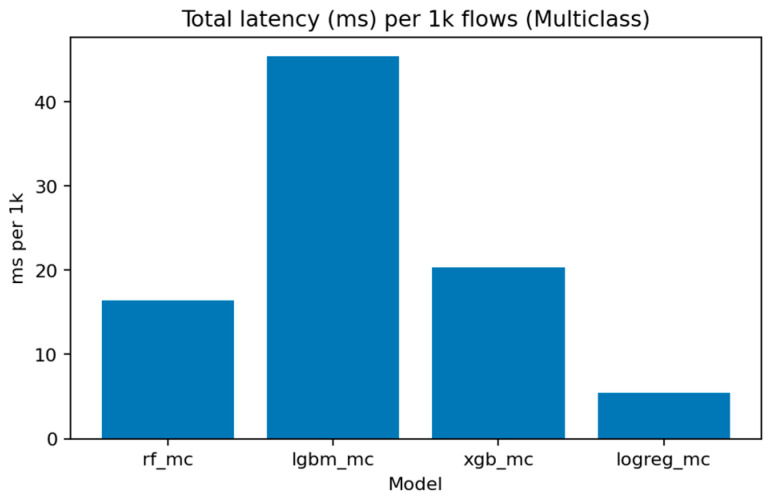
Total Inference Latency per 1k Samples (Multiclass).

**Figure 32:**
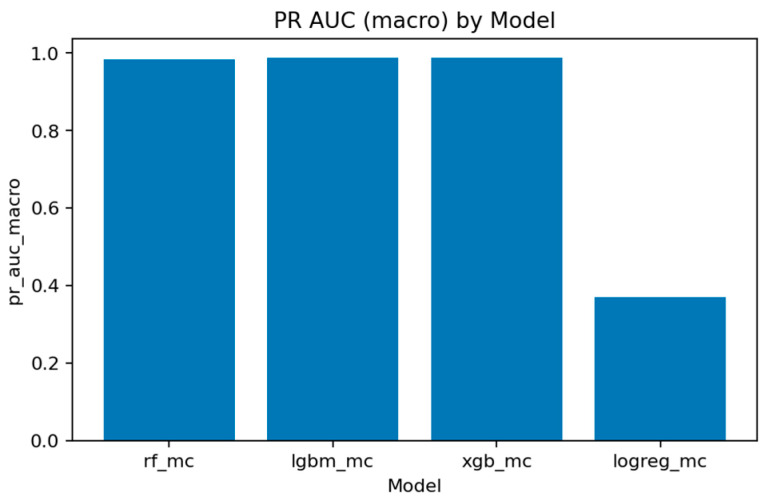
PR-AUC Macro.

**Figure 33:**
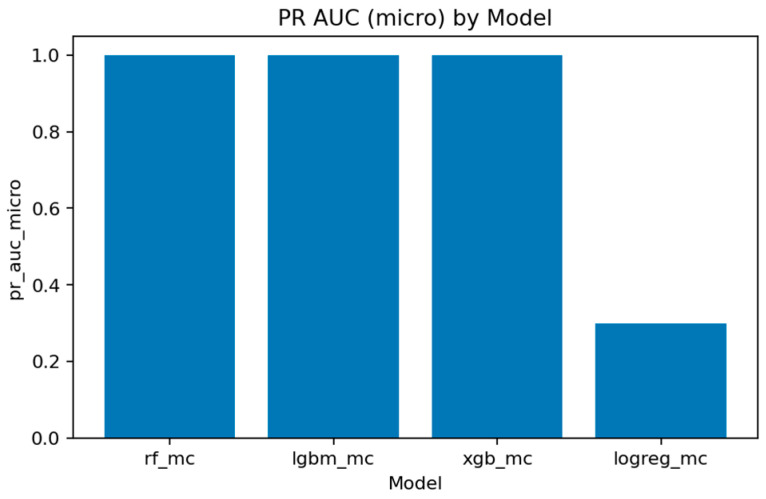
PR-AUC Micro.

**Figure 34:**
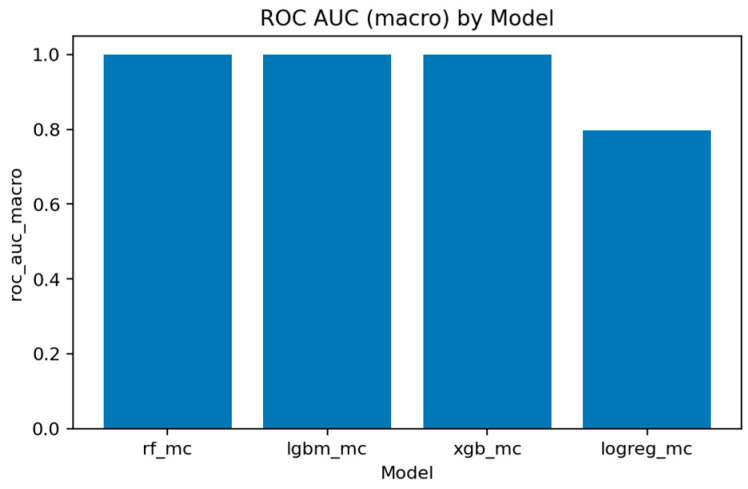
ROC-AUC Macro.

**Figure 35:**
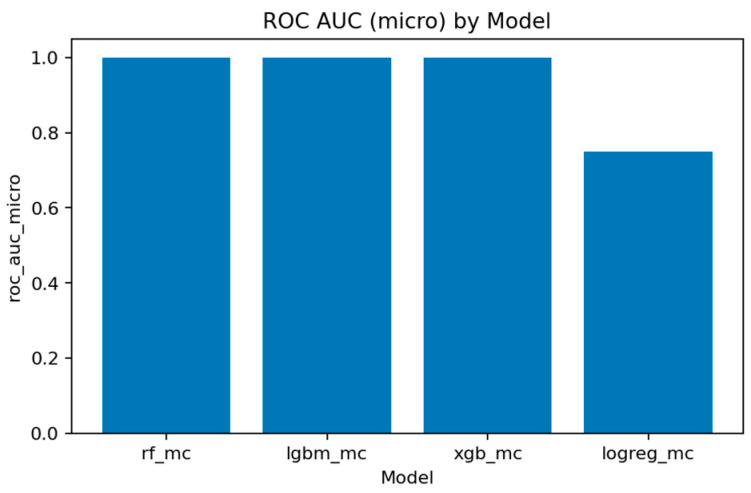
ROC-AUC Micro.

**Figure 36:**
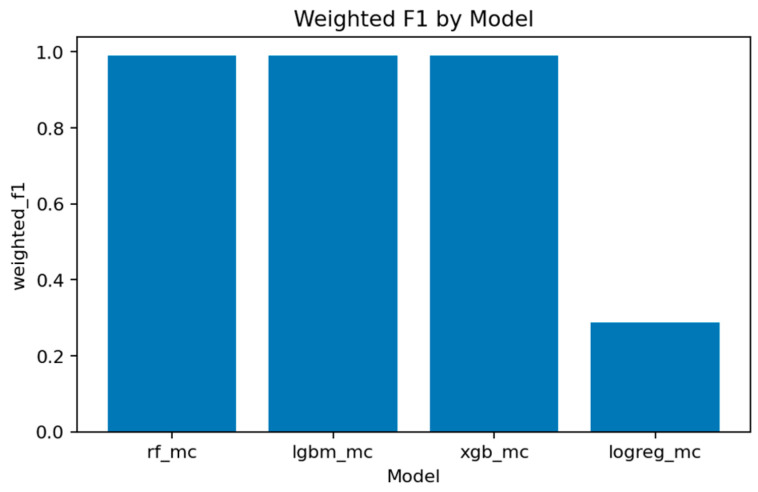
Weighted-F1.

**Figure 37:**
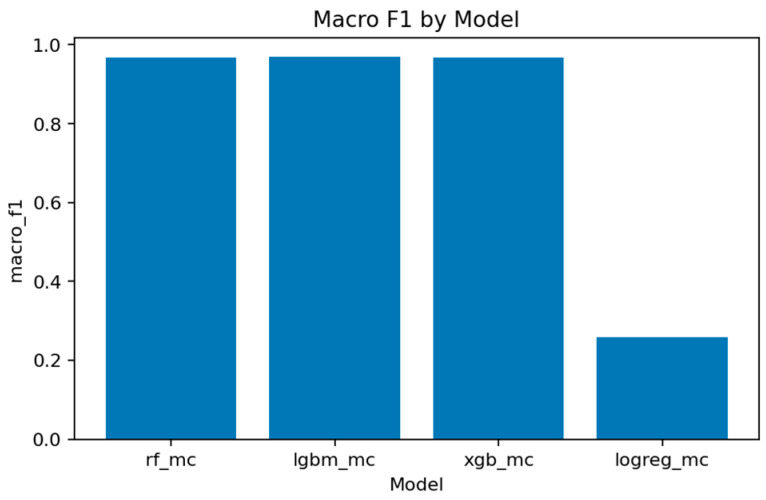
Macro-F1 Comparison.

A comprehensive comparison across all multiclass models is presented in Figure 31, Figure 32, Figure 33, Figure 34, Figure 35, Figure 36 and Figure 37 and Table 4, Table 5 and Table 6.

Figure 31 (Total Inference Latency per 1k Samples) compares execution times. Logistic Regression remains the fastest, processing 1000 flows in under 5 ms, while LightGBM and XGBoost require around 15–20 ms. Random Forest, though slightly slower, maintains competitive throughput for batch evaluations.

The multiclass LightGBM model exhibits substantially higher latency (~45.39 ms per 1K flows) compared to its binary counterpart (~10.03 ms per 1K flows), representing a 4.5× performance penalty. This increase is expected because the multiclass model uses more trees and evaluates multiple decision paths per instance to produce class probability scores for all ten labels. However, this latency gap suggests a natural deployment architecture for operational systems: (i) deploy a fast binary detector (LightGBM or XGBoost in binary mode) for real-time filtering at the network gateway, flagging suspicious flows with minimal delay; (ii) route flagged flows to a slower multiclass classifier running in batch mode on a monitoring server for detailed attack attribution and forensic analysis. This two-stage design maintains real-time responsiveness while preserving full attribution capability for incident response and threat intelligence.

Figure 32, Figure 33, Figure 34 and Figure 35 summarize the key quality metrics:

- Figure 32 and Figure 33 represent the area under the precision–recall curve aggregated across all classes. Macro averaging treats all classes equally, revealing the model’s fairness, while micro averaging weighs results by class frequency, emphasizing majority behavior.
- Figure 34 and Figure 35 illustrate overall discrimination capability. All ensemble models exceed 0.999 in both metrics, confirming stable separability across attack types.

**Figure 38:**
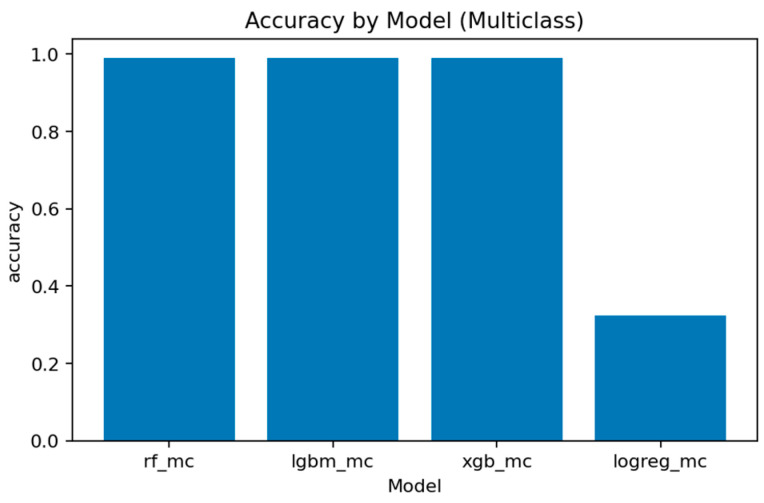
Accuracy Comparison (Multiclass).

Figure 36, Figure 37 and Figure 38 provide global accuracy and F1 comparisons:

- Figure 36 and Figure 37 demonstrate that LightGBM achieves the highest macro-F1 ≈ 0.969, closely followed by Random Forest and XGBoost. Weighted-F1 values are slightly higher, reflecting strong performance on dominant classes.
- Figure 38 consolidates final accuracy results, showing all ensemble models near 0.99, while the linear baseline remains substantially lower.

Table 4 summarizes latency, memory footprint, and model size from inference_benchmark_mc.csv. Table 5 (Per-Class Report—Aggregated) combines per-class F1, precision, and recall values from per_class_report_merged.csv, allowing direct comparison of sensitivity across attack types. Table 6 (Summary of Multiclass Models) presents consolidated metrics from summary_models_mc.csv, including overall accuracy, macro-F1, PR-AUC, ROC-AUC, and number of false predictions per class.

Overall, ensemble methods demonstrate exceptional multiclass performance with nearly identical accuracy and F1 results. The small discrepancies among LightGBM, XGBoost, and Random Forest fall within the margin of statistical noise. The main audit insight lies in the confusion structure: dos and ddos attacks remain difficult to separate due to near-identical flow signatures, while mitm requires additional temporal and session-level context to improve recognition. The models’ consistency across all metrics confirms their reliability as analytical tools for IoT traffic auditing and attack taxonomy analysis.

## 8. Discussion

The models demonstrate excellent performance on the most common attack types within the IoT/IIoT environment. Specifically, backdoor, denial-of-service (DoS), distributed denial-of-service (DDoS), scanning, injection, password, ransomware, and cross-site scripting (XSS) attacks are consistently detected with high accuracy and low false-negative rates. The ensemble methods, particularly LightGBM, Random Forest, and XGBoost, excel at distinguishing between these attacks and normal traffic, providing a reliable early-warning system for common IoT threats.

The strong performance on these frequent attack types suggests that the models are well-suited for large-scale deployments where these attacks are most likely to occur. Their ability to detect and classify these threats ensures that IoT networks can maintain high levels of operational integrity and security, with minimal resource overhead.

### 8.1. Risk Zone: Rare Mitm Attacks

The rare man-in-the-middle (MITM) attack type represents a notable risk zone in the audit findings. Despite the models’ high overall accuracy, the detection of MITM remains less reliable, especially given its relatively low occurrence in the training dataset. To address the class imbalance of the MITM class, we evaluated two standard mitigation methods: (i) class weighting and (ii) oversampling of minority instances. Applying class weights to the Random Forest and LightGBM models produced a consistent but modest improvement in detection performance (+0.05 percentage points F1-score for the MITM class, reducing false negatives and false positives by one instance each). Oversampling showed similar effects. These results indicate that the proposed framework can integrate imbalance-handling mechanisms with measurable benefits. However, the limited separability of MITM flow features in the TON_IoT dataset remains a core challenge, consistent with earlier reports in the literature [14,19].

The confusion matrix highlights frequent misclassifications of MITM as other attack types, particularly scanning or DoS-related attacks, due to their overlapping network characteristics.

Beyond class imbalance, we investigated the feature distributions for MITM flows and observed strong overlap with benign HTTP/TCP traffic in terms of duration, byte counts, and conn_state. In the TON_IoT Network dataset, MITM attacks appear to be manifested primarily at higher protocol layers (e.g., TLS certificates, HTTP payload manipulation), which are not available in our reduced feature set. We deliberately removed high-cardinality text fields (ssl_subject, ssl_issuer, http_uri, http_user_agent) to prevent leakage and enhance deployability, but these fields likely contain the distinguishing characteristics of MITM behavior. With flow-level features alone, the models cannot reliably separate MITM from normal web traffic, even when imbalance mitigation is applied. This represents a fundamental feature-related limitation rather than purely a sample size issue.

### 8.2. Practical Recommendations

Based on the audit findings, several practical security measures can be implemented to enhance network security, with each recommendation explicitly derived from our model analysis:

- Network Segmentation (motivated by: high importance of conn_state and duration features): Divide the network into smaller, isolated subnets to limit the spread of potential attacks. Given that our models identify REJ/S0 connection states as strong attack indicators, implement per-segment monitoring of abnormal connection state ratios. Establish baseline thresholds for REJ/S0 versus SF ratios per device, with automated alerts when devices exceed normal patterns.
- Rate-Limiting (motivated by: importance of byte count features): Implement rate-limiting mechanisms based on per-device traffic volume baselines. Since src_ip_bytes and dst_ip_bytes emerged as top predictive features, establish dynamic rate limits that account for typical IoT device behavior—for example, allowing higher sustained volumes for camera streams but flagging sudden spikes from sensors.
- MITM-Specific Controls (motivated by: persistent MITM detection failure despite flow-level features): Since MITM attacks remain indistinguishable using flow-level statistics alone (achieving only 78% F1 despite 99% overall accuracy), network operators should NOT rely solely on flow-based IDS for MITM detection. Instead, complement with TLS-level monitoring (certificate validation, unexpected cipher downgrades) and application-layer telemetry. Correlate network IDS alerts with gateway certificate logs and application authentication events.

These recommendations focus on proactive steps to secure IoT networks, making it harder for attackers to breach critical systems while facilitating faster detection and mitigation of potential threats.

### 8.3. Deployment Map and Efficiency Considerations

For effective deployment, models should be placed strategically within the network architecture. The key factors for model placement include latency, memory requirements, and the level of traffic analysis necessary.

- Edge Gateway: Lightweight models (such as Logistic Regression or small Random Forests) can be deployed on edge gateways for rapid traffic analysis and intrusion detection. These models provide real-time, low-latency detection of common attacks with minimal resource consumption.
- Monitoring Server: More complex models, such as LightGBM and XGBoost, can be hosted on dedicated monitoring servers. These models require more computational resources but offer enhanced accuracy and deeper insight into attack types across the network.

From the benchmark results, the latency per 1000 samples for models such as LightGBM and XGBoost is approximately 15–20 ms, making them suitable for centralized monitoring while maintaining acceptable throughput. The memory footprint for these models, however, may be higher compared to simpler models like Logistic Regression. This should be considered when deciding whether to deploy them on edge devices or central servers.

### 8.4. Ablations and Leakage Controls

Several measures were taken to ensure the robustness and fairness of the models, particularly regarding potential data leakage:

- Exclusion of High-Cardinality Text Fields: Features such as SSL certificates, HTTP user-agent strings, and session identifiers were excluded from the training process to prevent information leakage. Including such fields could lead to overfitting, where models “memorize” specific traffic patterns rather than learning generalizable attack behaviors.
- Feature Reduction: Dimensionality reduction techniques could be applied in future work to further decrease feature space and improve model efficiency, particularly in resource-constrained environments. However, careful validation is required to ensure that performance is not degraded.

Future ablation studies could explore:

- Impact of Service and Connection State Features: Investigating how excluding or including service-type fields (e.g., HTTP, FTP) and connection states (e.g., REJ, S0) affects model performance.
- Class Imbalance Mitigation: Experimenting with cost-sensitive learning techniques, such as focal loss or reweighting of minority classes, to improve performance on underrepresented attack types like MITM and XSS.
- Temporal Shifts: Evaluating models with time-based features or introducing time-series analysis to capture attack patterns that evolve over extended periods, such as slow DDoS or multi-stage intrusion attempts.

These changes could further enhance the models’ resilience and adaptability, particularly in dynamic IoT environments with evolving attack patterns.

### 8.5. Comparative Analysis with Prior Work

The performance of intrusion detection systems on the TON_IoT dataset has been extensively studied in the recent literature, yet comparing these results remains challenging due to variations in experimental setups, dataset splits, preprocessing strategies, and evaluation protocols. This section places the current audit findings within the broader landscape of IoT security research by examining methodological consistency, detection accuracy, and computational efficiency across multiple published studies.

Table 7 consolidates the main results from representative works that have evaluated their models on TON_IoT network data. The table includes binary and multiclass classification metrics where available, along with architectural details and computational characteristics. Several entries report only partial metrics due to the absence of standardized reporting practices in the field. Notably, very few studies publish model size, parameter counts, or per-sample inference times, making it difficult to assess deployment feasibility in resource-constrained IoT environments.

Several observations emerge from this comparison. First, the binary classification task on TON_IoT appears to be well-solved under controlled conditions, with multiple studies reporting accuracy above 99.5%. The ensemble models in the current audit (Random Forest, LightGBM, XGBoost) achieve results comparable to or exceeding the best reported values, with LightGBM reaching 99.93% accuracy. This consistency across different research groups suggests that the binary separation between normal and attack traffic contains strong discriminative signals that tree-based models can reliably exploit.

However, the apparent consensus on high accuracy masks important methodological differences. The original dataset study by Moustafa et al. (2021) [10] achieved 99.97% accuracy using GBM, but this result included IP addresses and port numbers as features—attributes that can introduce data leakage and overestimate real-world performance. The current audit explicitly excluded such high-cardinality identifiers to ensure that models generalize to unseen network configurations rather than memorizing specific connections. When this precaution is applied, accuracy remains high, but the detection task becomes more challenging, as reflected in the increased false negative rates for rare attack types like MITM. Notably, all three ensemble models in this audit achieve perfect or near-perfect ROC-AUC (1.000) and PR-AUC (1.000) on binary classification, confirming strong discrimination capability even with conservative feature engineering.

The multiclass classification results reveal greater variation. DIS-IoT by Lazzarini et al. (2023) [19] reports the highest multiclass accuracy at 99.7%, though the paper does not specify whether features like IP addresses were excluded. The current audit’s LightGBM model achieves 99.03% accuracy with precision of 98.95%, recall of 98.75%, weighted F1 of 99.04%, and macro-F1 of 96.94%, placing it among the top-performing methods while maintaining strict feature hygiene. The macro-averaged PR-AUC of 98.76% further confirms balanced performance across all attack classes. Notably, the macro-F1 metric—which treats all classes equally regardless of frequency—provides a fairer assessment of model robustness than raw accuracy, especially given the severe class imbalance in TON_IoT (e.g., MITM represents only 0.5% of samples). By this measure, LightGBM outperforms most published results that report macro-averaged metrics. Random Forest and XGBoost achieve comparable multiclass performance (accuracy 98.97% and 99.02%, macro-F1 96.81% and 96.80%, respectively), demonstrating consistency across ensemble architectures.

One striking gap in the literature is the near-total absence of efficiency benchmarks. Only Wang et al. (2024) [16] report computational complexity in terms of FLOPs and MAdds, and no prior work publishes per-sample inference times or model sizes in a form useful for deployment planning. The current audit addresses this gap by systematically measuring inference latency and serialized model size for all trained classifiers. LightGBM processes 1000 binary predictions in approximately 10 milliseconds on CPU, with a footprint of 2.76 MB—small enough to deploy on gateway hardware with modest resources. XGBoost offers even lower latency (6.57 ms per 1k samples) at the cost of slightly reduced accuracy. These measurements suggest that high detection accuracy need not come at the expense of real-time responsiveness, a critical consideration for operational IoT security systems.

The comparison also highlights the value of baseline models. Logistic Regression, included here as a linear reference, achieves only 87.2% accuracy in binary mode (with precision and recall both at 91.46%) and collapses to 32.3% accuracy in multiclass mode (precision 47.84%, recall 25.64%, macro-F1 25.90%). This dramatic performance drop confirms that the classification problem is fundamentally nonlinear and that ensemble methods genuinely provide added value rather than simply overfitting to test data. The weak performance of linear models stands in contrast to the strong results from tree-based ensembles, reinforcing the interpretability–efficiency trade-off inherent in IoT intrusion detection.

It is worth noting that several studies reformulated the TON_IoT classification problem to reduce the number of classes. For example, Ammar et al., 2025 [9] merged the original ten labels into five broader categories (normal, dos_ddos, web_attacks, malware, other_attacks), achieving 96% ROC-AUC on this simplified task. While such aggregations can improve minority class detection, they sacrifice the granularity needed for precise threat attribution—a key requirement in security audit contexts where operators must prioritize responses based on attack type. The current work retains the full ten-class structure to preserve this diagnostic capability.

Another methodological consideration concerns dataset splits and cross-validation strategies. Some studies use stratified k-fold validation, while others rely on single train–test splits. The current audit employs a fixed 80/20 stratified split with a deterministic random seed, ensuring that results can be exactly reproduced. This approach trades the robustness of cross-validation for the transparency of a single, publicly documented partition. Given that the test set contains over 42,000 samples, the variance due to split selection is likely negligible, but the choice reflects a broader tension in reproducibility practices between statistical rigor and practical replicability.

Finally, the comparison reveals an intriguing pattern: newer models do not consistently outperform earlier work. DLTIF (Kumar et al., 2023 [27]) and DIS-IoT (Lazzarini et al., 2023 [19]) achieve results that match or exceed more recent proposals, suggesting that the performance ceiling on TON_IoT may have been reached under current feature engineering paradigms. Further improvements likely require either richer feature representations (e.g., temporal sequences, graph-structured data) or better handling of minority classes through techniques like cost-sensitive learning or synthetic data augmentation. The current audit’s finding that MITM detection remains problematic across all models supports this interpretation—high overall accuracy conceals persistent weaknesses in rare attack recognition.

In summary, the ensemble models evaluated in this audit perform competitively with the best published results on TON_IoT while providing transparency in feature selection, computational cost, and reproducibility infrastructure that is often absent from prior work. The systematic reporting of inference times, model sizes, and complete metric sets (including per-class precision, recall, and F1 scores for all ten attack categories) offers a practical foundation for deployment decisions, and the consistent evaluation protocol enables fair comparison across model families. The achievement of near-perfect ROC-AUC scores (≥0.9995) across all ensemble models in both binary and multiclass settings confirms the reliability of these approaches for IoT security auditing. However, the ceiling effects observed in binary classification and the persistent challenges in rare class detection (particularly MITM, with F1 scores around 77–78% despite overall accuracy above 99%) suggest that incremental improvements within the current paradigm may be reaching diminishing returns. Future work should explore alternative data representations and class-balancing strategies to address the remaining blind spots in IoT intrusion detection.

## 9. Conclusions

This security audit framework evaluated the defensive capabilities of IoT device networks through systematic assessment of machine learning-based intrusion detection. By applying transparent methodology, comprehensive benchmarking, and reproducible experimental protocols to the TON_IoT dataset, we demonstrated how network traffic analysis can inform security posture evaluation while meeting operational deployment requirements.

The audit findings reveal both strengths and limitations in current detection capabilities. Binary classification—the ability to distinguish compromised traffic from legitimate operations—performs reliably across all ensemble models. Random Forest, LightGBM, and XGBoost each achieve accuracy exceeding 99.8% with perfect ROC-AUC (1.000) and inference times under 12 milliseconds per 1000 flows. These results confirm that fundamental threat detection is operationally viable: IoT networks can be monitored in near-real-time using models that fit on resource-constrained gateway hardware. LightGBM provides the optimal balance for deployment, combining 99.93% detection accuracy with a 2.76 MB footprint and 10 ms processing latency—specifications that enable edge-based monitoring without centralized infrastructure dependencies.

Multiclass threat attribution exposes more complex audit findings. While overall accuracy remains high (99.0% for LightGBM with macro-F1 of 96.94%), per-class analysis reveals systematic blind spots that aggregate metrics conceal. Man-in-the-middle attacks—among the most serious threats to IoT device communications—achieve only 78% F1 scores despite representing clear security risks. This discrepancy between overall performance and minority class detection illustrates a critical audit principle: accuracy alone cannot assess security posture when threat distributions are imbalanced. Organizations evaluating their defensive capabilities must examine per-class confusion matrices and macro-averaged metrics to understand which attacks their monitoring systems reliably detect versus which remain invisible.

The comparative analysis positions these findings within the broader research landscape. Our models match or exceed the best published accuracy on TON_IoT while uniquely providing the transparency required for security audit: computational benchmarks, per-class error analysis, complete reproducibility artifacts, and explicit documentation of feature engineering decisions. Twenty reviewed studies report detection accuracy but few disclose inference times, model sizes, or minority class performance—the information security assessors need to evaluate deployment feasibility and understand detection limitations. This gap between algorithmic evaluation and audit requirements reflects a fundamental misalignment: research optimizes for leaderboard metrics while practitioners need credible risk assessment.

The audit methodology enforced several constraints designed to prevent overfitting and ensure generalization. Exclusion of high-cardinality features (IP addresses, session identifiers, user agents) prevents models from memorizing specific network configurations rather than learning attack signatures. Fixed train–test splits with deterministic seeds enable exact replication of results. Serialized preprocessing pipelines ensure consistent feature transformations. These constraints may sacrifice marginal accuracy gains but deliver the reproducibility and transparency that credible security audits demand. The framework demonstrates that rigorous methodology and competitive performance are not mutually exclusive.

Several limitations constrain the scope of audit conclusions. The evaluation uses a single dataset (TON_IoT) captured in controlled testbed conditions, which may not reflect attack distributions in production environments. Flow-level feature aggregation discards temporal sequences and payload details that could improve detection of sophisticated multi-stage attacks. The models treat each flow independently, ignoring session context and device behavior history that operational systems could leverage. And the experimental protocol uses a single train–test split rather than cross-validation, trading statistical robustness for exact reproducibility.

Future audit methodologies should address these gaps through complementary extensions. Temporal modeling using sequence-based architectures could capture attack patterns that unfold across multiple flows, improving detection of reconnaissance and lateral movement. Feature enrichment with application-layer signals (HTTP headers, TLS handshake metadata, DNS query patterns) might reduce blind spots in man-in-the-middle and injection attack detection. Class-balancing techniques including focal loss and synthetic minority oversampling could improve rare threat detection without degrading overall accuracy. Adversarial robustness evaluation would assess whether detection systems degrade gracefully when attackers actively evade monitoring. And multi-dataset validation would confirm that audit findings generalize across different IoT deployment scenarios.

The fundamental goal of security auditing is risk assessment—understanding what threats a defensive system can detect, which it might miss, and what mitigations address the gaps. Traditional machine learning evaluation focuses on aggregate accuracy, treating misclassifications as symmetric errors. Security audits require a different perspective: false negatives for critical threats (man-in-the-middle, backdoor installation) carry higher consequences than false positives for low-risk events (port scanning). Our framework incorporates this asymmetry through per-class analysis, confusion matrix examination, and explicit mapping from detection failures to recommended mitigations (network segmentation, rate-limiting, TLS inspection).

The practical value of this audit framework extends beyond the specific models evaluated. The methodology—transparent feature engineering, computational benchmarking, per-class error analysis, reproducibility artifacts—can be applied to any intrusion detection system targeting IoT device networks. Organizations conducting security assessments can use these techniques to evaluate vendor-provided monitoring tools, validate claimed detection rates, and identify blind spots that require compensating controls. Researchers can adopt the experimental protocols to enable fair comparison across studies and accelerate progress toward deployable security solutions.

The significance of this work extends beyond the specific models evaluated. By demonstrating that rigorous methodology and competitive performance are compatible, we provide a template for credible IoT security assessment that satisfies both research standards and operational requirements. Organizations conducting security audits can adopt these techniques to evaluate vendor claims, identify blind spots, and prioritize compensating controls. Researchers can use the reproducible baseline to fairly measure the added value of novel architectures.

Security audits ultimately serve decision-makers who must balance risk, cost, and operational complexity. A model with 99% accuracy sounds impressive but provides little actionable information. Does it run on edge hardware or require cloud infrastructure? Does it detect all attack types equally or excel at common threats while missing rare ones? Can its performance claims be independently verified? The audit framework presented here ensures these questions have documented answers, transforming abstract accuracy metrics into concrete risk assessments that inform deployment decisions and security investments. Whether the resulting defensive posture proves adequate depends on organizational threat models and risk tolerances that only practitioners can assess. Our contribution is ensuring those assessments rest on complete, verifiable, and reproducible evidence.

## Figures and Tables

**Table 1:** Label distribution in the TON_IoT Network Dataset.

Label	Samples	Share (%)
normal	50,000	23.7
backdoor	21,035	10.0
ddos	23,000	10.9
dos	22,000	10.4
injection	20,500	9.7
mitm	1043	0.5
password	21,700	10.3
ransomware	21,800	10.3
scanning	20,900	9.9
xss	9065	4.3

**Table 2:** Inference Benchmark (measured on AMD Ryzen 7 7840HS, 64 GB RAM, Windows 11).

Model	Transform Time (ms/1K)	Prediction Time (ms/1K)	Total Latency (ms/1K)	Samples
RF	3.8303	7.2994	11.1296	10,000
LGBM	5.5334	4.4990	10.0324	10,000
XGB	5.5568	1.0091	6.5658	10,000
LOGREG	5.1382	0.2006	5.3387	10,000

**Table 3:** Summary of Binary Model Metrics.

Model	RF	LGBM	XGB	LOGREG
Accuracy	0.9989	0.9993	0.9989	0.8720
F1 (Attack)	0.9993	0.9995	0.9993	0.9146
F1 (Normal)	0.9976	0.9985	0.9976	0.7452
ROC-AUC	1.0000	1.0000	1.0000	0.9213
PR-AUC	1.0000	1.0000	1.0000	0.9706
FP	31	11	24	2102
FN	16	20	24	3299
Model Size (MB)	23.7390	2.7480	0.9330	0.0020
Preproc. Size (MB)	0.0090	0.0090	0.0090	0.0090
Total Size (MB)	23.7480	2.7570	0.9410	0.0100

**Table 4:** Inference Benchmark—Multiclass (measured on AMD Ryzen 7 7840HS, 64 GB RAM, Windows 11).

Model	Transform Time (ms/1K)	Prediction Time (ms/1K)	Total Latency (ms/1K)	Samples
RF_MC	3.5316	12.8546	16.3863	10,000
LGBM_MC	3.9658	41.4263	45.3921	10,000
XGB_MC	5.0596	15.2601	20.3197	10,000
LOGREG_MC	5.0404	0.4011	5.4415	10,000

**Table 5:** Per-Class Report—Aggregated.

**Class**	**Precision RF_MC**	**Recall RF_MC**	**F1 RF_MC**	**Precision LGBM_MC**	**Recall LGBM_MC**	**F1 LGBM_MC**
backdoor	1.0000	0.9993	0.9996	1.0000	0.9993	0.9996
ddos	0.9857	0.9835	0.9846	0.9895	0.9875	0.9885
dos	0.9864	0.9823	0.9843	0.9875	0.9840	0.9857
injection	0.9771	0.9703	0.9737	0.9800	0.9693	0.9746
mitm	0.7403	0.8182	0.7773	0.7154	0.8660	0.7835
normal	0.9998	1.0000	0.9999	1.0000	1.0000	1.0000
password	0.9950	0.9930	0.9940	0.9960	0.9915	0.9937
ransomware	1.0000	0.9990	0.9995	1.0000	0.9990	0.9995
scanning	0.9875	0.9903	0.9889	0.9895	0.9873	0.9884
xss	0.9755	0.9838	0.9796	0.9734	0.9870	0.9801
**Class**	**Precision XGB_MC**	**Recall XGB_MC**	**F1 XGB_MC**	**Precision LOGREG_MC**	**Recall LOGREG_MC**	**F1 LOGREG_MC**
backdoor	1.0000	0.9993	0.9996	0.0000	0.0000	0.0000
ddos	0.9877	0.9855	0.9866	0.0956	0.0098	0.0177
dos	0.9864	0.9825	0.9845	0.0000	0.0000	0.0000
injection	0.9858	0.9685	0.9770	0.6603	0.6735	0.6668
mitm	0.7208	0.8278	0.7706	0.0277	0.2679	0.0503
normal	1.0000	1.0000	1.0000	0.4784	0.2564	0.3339
password	0.9945	0.9915	0.9930	0.6727	0.3838	0.4887
ransomware	1.0000	0.9990	0.9995	0.3341	0.1440	0.2013
scanning	0.9868	0.9895	0.9881	0.4463	0.8040	0.5740
xss	0.9720	0.9895	0.9807	0.1554	0.7418	0.2569

**Table 6:** Summary of Multiclass Models.

Model	RF_MC	LGBM_MC	XGB_MC	LOGREG_MC
Accuracy	0.9897	0.9903	0.9902	0.3233
Macro-F1	0.9681	0.9694	0.9680	0.2590
Weighted-F1	0.9898	0.9904	0.9902	0.2883
ROC-AUC (Micro)	0.9996	0.9999	1.0000	0.7486
ROC-AUC (Macro)	0.9995	0.9998	0.9998	0.7971
PR-AUC (Micro)	0.9987	0.9995	0.9996	0.2995
PR-AUC (Macro)	0.9824	0.9876	0.9869	0.3698
Model Size (MB)	250.0690	29.3010	12.4540	0.0100
Preproc. Size (MB)	0.0080	0.0080	0.0080	0.0080
Total Size (MB)	250.0780	29.3090	12.4620	0.0190

**Table 7:** Comparative Performance on TON_IoT Network Dataset.

Source	Task Type	Methodology	Accuracy	Precision	Recall	F1-Score	ROC-AUC	Macro-F1	Model Size (MB)	Inference Time (ms/1K)	Notes
Moustafa, 2021 [10]	Binary	GBM	0.9997	0.9998	0.9997	0.9997	--	--	--	--	Dataset authors; includes IP/port features
Moustafa, 2021 [10]	Binary	Random Forest	0.9986	0.9985	0.9988	0.9986	--	--	--	--	ntrees = 150
Moustafa, 2021 [10]	Binary	Deep NN	0.9959	0.9960	0.9957	0.9958	--	--	--	--	15 hidden layers
Moustafa, 2021 [10]	Binary	Naive Bayes	0.8936	0.9092	0.8936	0.8835	--	--	--	--	Baseline
Mishra et al., 2024 [18]	Binary	Ensemble model	0.9698	--	--	--	--	--	--	--	Discrepancy in paper: text reports 99.98%
Keshk et al., 2023 [14]	Binary	LSTM	0.9789	0.9812	0.9765	0.9788	--	--	--	--	Information Sciences
Keshk et al., 2023 [14]	MC	LSTM	0.9534	0.9558	0.9510	0.9533	--	--	--	--	10 attack types
Kumar et al., 2023 [27]	Binary	DLTIF	0.9990	--	--	--	--	--	--	--	Best binary accuracy in review
Kumar et al., 2023 [27]	MC	DLTIF	0.9930	--	--	0.9930	--	--	--	--	Deep learning framework
Lazzarini et al., 2023 [19]	Binary	DIS-IoT	0.9960	--	--	--	1.0000	--	--	--	Only work reporting AUC
Lazzarini et al., 2023 [19]	MC	DIS-IoT	0.9970	0.9970	0.9970	0.9970	--	--	--	--	Best multiclass in the literature
Al-Wesabi et al., 2023 [12]	Binary	POAFL-DDC	0.9956	--	--	--	--	--	--	--	30% test split
Escorcia-Gutierrez et al., 2023 [13]	MC	STFA-HDLID	0.9951	--	--	--	--	--	--	--	10,000 samples, 10 classes
Kale & Thing, 2023 [17]	Binary	DevNet	--	--	1.0000	--	--	--	--	--	Windows 10 subset only
Jia et al., 2025 [15]	MC	IDEAL	--	0.9870	0.9890	--	--	0.9880	--	--	Macro-averaged metrics
Wang et al., 2024 [16]	MC	BT-TPF	0.9920	--	--	0.9920	--	--	--	--	FLOPs: 4.1 × 10^3^, MAdds: 8.2 × 10^3^
Hwang et al., 2026 [5]	MC	CG-LLM	0.9480	--	--	--	0.9972	0.9193	--	--	Graph NN + TinyLlama LLM
Campos et al., 2022 [11]	Binary	Federated Learning	~0.98–0.99	--	--	--	--	--	--	--	4–10 parties, approx. from graphs
Ammar et al., 2025 [9]	MC	GOA-CNN	--	--	--	--	0.9600	--	--	--	5-class reformulation
Wu et al., 2025 [28]	Binary	FSMMTD	0.9890	0.9900	0.9880	0.9890	--	--	--	--	TON_IoT + CICIoV2024 combined
**This Work**	**Binary**	**Random Forest**	**0.9989**	**0.9993**	**0.9993**	**0.9993**	**1.0000**	**--**	**23.74**	**11.13**	**PR-AUC: 1.000; FN: 16**
**This Work**	**Binary**	**LightGBM**	**0.9993**	**0.9995**	**0.9995**	**0.9995**	**1.0000**	**--**	**2.75**	**10.03**	**PR-AUC: 1.000; FN: 20**
**This Work**	**Binary**	**XGBoost**	**0.9989**	**0.9993**	**0.9993**	**0.9993**	**1.0000**	**--**	**0.93**	**6.57**	**PR-AUC: 1.000; FN: 24**
**This Work**	**Binary**	**Logistic Regression**	**0.8720**	**0.9146**	**0.9146**	**0.9146**	**0.9213**	**--**	**0.002**	**5.34**	**PR-AUC: 0.971; FN: 3299**
**This Work**	**MC**	**Random Forest**	**0.9897**	**0.9857**	**0.9835**	**0.9898**	**0.9995**	**0.9681**	**250.08**	**16.39**	**PR-AUC (macro): 0.982**
**This Work**	**MC**	**LightGBM**	**0.9903**	**0.9895**	**0.9875**	**0.9904**	**0.9998**	**0.9694**	**29.31**	**45.39**	**PR-AUC (macro): 0.988**
**This Work**	**MC**	**XGBoost**	**0.9902**	**0.9877**	**0.9855**	**0.9902**	**0.9998**	**0.9680**	**12.46**	**20.32**	**PR-AUC (macro): 0.987**
**This Work**	**MC**	**Logistic Regression**	**0.3233**	**0.4784**	**0.2564**	**0.2883**	**0.7971**	**0.2590**	**0.02**	**5.44**	**PR-AUC (macro): 0.370**

Note: For binary classification, Precision, Recall, and F1-Score columns report positive class (attack) metrics. For multiclass classification (MC), these columns report macro-averaged values to enable comparison across studies with different reporting conventions. ROC-AUC values for multiclass tasks represent micro-averaged scores unless otherwise noted. Complete per-class metrics for the current work are available in the source tables.

## Data Availability

The complete codebase for this research, including data processing, model implementation, and visualization scripts, is freely available at https://github.com/KuznetsovKarazin/iot-audit (accessed on 6 November 2025). This accessibility enables direct verification of our results and facilitates further extension of our work by interested researchers.

## Appendix A. Feature Engineering Details

### Appendix A.1. Preprocessing Code Structure

The feature engineering pipeline is implemented in src/iot_audit/preprocessing.py with the following logic:

- Numerical features: All columns with numeric dtype (int, float) are retained.
- Categorical features: Explicitly whitelisted low-cardinality columns plus auto-detected non-numeric columns with ≤40 unique values.
- Excluded columns: High-cardinality text fields that risk data leakage.
- One-hot encoding: Applied WITHOUT drop parameter, meaning k distinct values produce k binary columns.

### Appendix A.2. Feature Categories

**Table A1:** Feature Processing Summary.

Feature Type	Count	Processing Method
Numerical features	23	Median imputation only (no scaling in baseline)
Categorical features (before encoding)	6–9	Most-frequent imputation → OneHotEncoder
One-hot encoded binary features	~13	Depends on distinct values in training set

### Appendix A.3. Numerical Features

These features are used as-is after median imputation (Table A2).

**Table A2:** Numerical Features (23 features).

Feature Name	Description	Typical Range
src_port	Source port number	0–65,535
dst_port	Destination port number	0–65,535
duration	Flow duration in seconds	0.0–∞
src_bytes	Total bytes sent from source	0–∞
dst_bytes	Total bytes sent to destination	0–∞
missed_bytes	Bytes missed during capture	0–∞
src_pkts	Total packets sent from source	0–∞
src_ip_bytes	IP-layer bytes from source	0–∞
dst_pkts	Total packets sent to destination	0–∞
dst_ip_bytes	IP-layer bytes to destination	0–∞
dns_qclass	DNS query class (numeric code)	0–65,535
dns_qtype	DNS query type (numeric code)	0–65,535
dns_rcode	DNS response code (numeric)	0–15
dns_AA	DNS authoritative answer flag	{0, 1}
dns_RD	DNS recursion desired flag	{0, 1}
dns_RA	DNS recursion available flag	{0, 1}
dns_rejected	DNS query rejected flag	{0, 1}
ssl_resumed	SSL session resumed flag	{0, 1}
ssl_established	SSL handshake completed flag	{0, 1}
http_trans_depth	HTTP pipelined transaction depth	0–∞
http_request_body_len	HTTP request body length	0–∞
http_response_body_len	HTTP response body length	0–∞
http_status_code	HTTP status code (numeric)	100–599

Note: dns_qtype, dns_rcode, and http_status_code are stored as numeric codes in the dataset, so they are treated as numerical features despite appearing in CATEGORICAL_SAFE (the numeric dtype takes precedence).

### Appendix A.4. Categorical Features → One-Hot Encoded

**Table A3:** Categorical Features and One-Hot Expansion.

Original Feature	Typical Distinct Values	Approx. One-Hot Columns	Example Categories
proto	3	3	tcp, udp, icmp
service	2–5	2–5	http, dns, -, ssh, ftp
conn_state	3–5	3–5	SF, S0, REJ, S1, RSTO
ssl_version	2–3	2–3	TLSv12, TLSv13, -
http_method	2–3	2–3	GET, POST, -
weird_name	1–2	1–2	Various Zeek anomaly types or -
Possible extras	-	0–3	ssl_cipher, http_version, weird_notice (if ≤40 unique)

Note: Total one-hot encoded features: ~13 binary columns.

Explanation of low cardinality:

- Many categorical fields contain primarily missing values (encoded as “-” or NaN).
- TON_IoT network flows are dominated by a small subset of protocols (TCP) and services (HTTP, DNS).
- OneHotEncoder creates one column per distinct value INCLUDING the missing indicator.
- For example, if service has only {http, dns, -}, it produces exactly 3 binary columns.

### Appendix A.5. Excluded High-Cardinality Fields

**Table A4:** Fields Excluded to Prevent Data Leakage.

Excluded Field	Reason
src_ip	High cardinality; memorization of specific hosts
dst_ip	High cardinality; memorization of specific hosts
ssl_subject	Certificate subject DN (organization-specific strings)
ssl_issuer	Certificate issuer DN (CA-specific strings)
http_uri	Full URIs with session IDs and query parameters
http_user_agent	Browser/client identification strings
http_orig_mime_types	Concatenated MIME type lists
http_resp_mime_types	Concatenated MIME type lists
weird_addl	Free-form text anomaly descriptions
dns_query	Full domain names with unique subdomains

Note: These fields are silently dropped by the ColumnTransformer (remainder = “drop”).
